# Thymoquinone Prevents Dopaminergic Neurodegeneration by Attenuating Oxidative Stress Via the Nrf2/ARE Pathway

**DOI:** 10.3389/fphar.2020.615598

**Published:** 2021-01-14

**Authors:** Jianjian Dong, Xiaoming Zhang, Shijing Wang, Chenchen Xu, Manli Gao, Songyang Liu, Xiaoxiao Li, Nan Cheng, Yongsheng Han, Xun Wang, Yongzhu Han

**Affiliations:** ^1^ High Magnetic Field Laboratory, Hefei Institutes of Physical Science, Chinese Academy of Sciences, Hefei, China; ^2^ University of Science and Technology of China, Hefei, China; ^3^ The Affiliated Hospital of the Neurology Institute, Anhui University of Chinese Medicine, Hefei, China; ^4^ Anhui University of Chinese Medicine, Hefei, China

**Keywords:** Parkinson’ s disease, thymoquinone, Nrf2/ARE signaling pathway, oxidative stress, neurodegeneration

## Abstract

Studies have indicated that oxidative stress plays a crucial role in the development of Parkinson’s disease (PD) and other neurodegenerative conditions. Research has also revealed that nuclear factor erythroid 2-related factor 2 (Nrf2) triggers the expression of antioxidant genes via a series of antioxidant response elements (AREs), thus preventing oxidative stress. Thymoquinone (TQ) is the bioactive component of *Nigella sativa*, a medicinal plant that exhibits antioxidant and neuroprotective effects. In the present study we examined whether TQ alleviates *in vivo* and *in vitro* neurodegeneration induced by 1-methyl-4-phenylpyridinium (MPP^+^) and 1-methyl-4-phenyl-1,2,3,6-tetrahydropyridine (MPTP) by acting as an activator of the Nrf2/ARE cascade. We showed that TQ significantly reduced MPP^+^-mediated cell death and apoptosis. Moreover, TQ significantly elevated the nuclear translocation of Nrf2 and significantly increased the subsequent expression of antioxidative genes such as Heme oxygenase 1 (HO-1), quinone oxidoreductase (NQO1) and Glutathione-S-Transferase (GST). The application of siRNA to silence Nrf2 led to an abolishment in the protective effects of TQ. We also found that the intraperitoneal injection of TQ into a rodent model of PD ameliorated oxidative stress and effectively mitigated nigrostriatal dopaminergic degeneration by activating the Nrf2-ARE pathway. However, these effects were inhibited by the injection of a lentivirus wrapped Nrf2 siRNA (siNrf2). Collectively, these findings suggest that TQ alleviates progressive dopaminergic neuropathology by activating the Nrf2/ARE signaling cascade and by attenuating oxidative stress, thus demonstrating that TQ is a potential novel drug candidate for the treatment of PD.

## Introduction

Parkinson’s disease (PD) is a non-reversible and age-linked chronic neurodegenerative condition that is typified by the depletion of nigrostriatal dopaminergic neurons. The resulting depletion of dopamine causes resting tremor, postural instability, rigidity, and bradykinesia. Although the precise cause of PD has yet to be elucidated, an accumulating body of evidence suggests that oxidative stress has a significant influence on the pathogenesis of PD (Grayson, 2016). Multiple biomechanisms have been proposed to affect the mitochondria of dopaminergic neurons, result in increased production of reactive oxygen species (ROS) (Schapira and Jenner, 2011). ROS can result in covalent oxidative modifications such as the oxidation of RNA and can induce mutations in mitochondrial DNA (mtDNA), thus affecting the stability of nucleic acids (Angelova and Abramov, 2018). Moreover, oxidative modifications are known to interfere with protein homeostasis by expediting the aggregation of α-synuclein and parkin, and by dissociating the proteasome (Scudamore and Ciossek, 2018). These modifications may cause cellular dysfunction and even apoptosis. The mitochondrial-dependent caspase pathway is known to play an essential role in apoptosis (Van Opdenbosch and Lamkanfi, 2019). Research has shown that the stimulation of this cascade induces the release of proapoptotic factors into the cytosol, including cytochrome c (Cyc), activating caspase-9, and caspase-3; thus, triggering cellular apoptosis (Green and Llambi, 2015). Therefore, it is evident that the antioxidant pathways that regulate mechanisms to ameliorate oxidative damage may also exhibit neuroprotective effects (Izumi et al., 2018).

The most significant of the endogenous antioxidant cascades is the Nrf2/ARE signaling cascade (Buendia et al., 2016). When exposed to ROS or other exogenous toxicants, the cytoplasmic NFE-related factor 2 (Nrf2) becomes activated, disengages from kelch-like ECH-associated protein (Keap1) and translocates into the nucleus. Within the nucleus, activated Nrf2 then interacts with antioxidant reactive elements (AREs) which have been shown to be distinct regulators of antioxidant molecules (Zhang et al., 2016). Subsequently, ARE-mediated processes induce the activation of a range of antioxidative enzymes and detoxifying enzymes, including heme oxygenase 1 (HO-1), quinone oxidoreductase (NQO1), NAD(P)H, and glutathione-S-transferase (GST). Collectively, these factors play critical roles in sustaining cellular function (Buendia et al., 2016). An accumulating body of evidence now suggests that the Nrf2-ARE cascade plays a significant role in the development of PD (Fão et al., 2019; Gureev and Popov, 2019). For example, clinical studies have reported that patients with PD showed reduced expression levels of 31 genes that contained the ARE-sequence in their promoters; these patients also expressed increased levels of Nrf2 (Wang et al., 2017). Moreover, research has shown that the Nrf2-ARE axis forms a crucial antioxidant defense pathway that demonstrated neuroprotective effects in an experimental model of PD by inhibiting oxidative stress and neuroinflammation (Wang et al., 2017). Collectively, these findings indicate that drugs that activate Nrf2 could impede the pathogenesis of PD.

Thymoquinone (TQ) is the primary active ingredient of the vaporous oil from *Nigella sativa* and is known to exhibit antioxidant and anti-inflammatory properties (Darakhshan et al., 2015). A number of studies have demonstrated that TQ can exert effects against apoptosis in primary dopaminergic cells triggered by exposure to 1-methyl-4-phenylpyridinium (MPP^+^) and rotenone (Radad et al., 2009; Radad et al., 2015). Another study reported that TQ specifically averted rotenone-triggered motor defects and variations in the levels of TH, dynamin-related protein-1(Drp1), dopamine, and Parkin (Ebrahimi et al., 2017). In the Hemi-Parkinson rat model, induced by exposure to 6-hydroxydopamine (6-OHDA), TQ has been reported to reduce the levels of malondialdehyde (MDA) and prevent the degeneration of dopaminergic neurons, thus suggesting that TQ has an antioxidant effect (Sedaghat et al., 2014). Evidence therefore indicates that oxidative stress, as well as dysregulation of the Nrf2-ARE signaling cascades, plays a key role in the development of PD (Gureev and Popov, 2019). However, a precise mechanism that links TQ, oxidative stress, and the Nrf2-ARE signaling cascades, has yet to be identified in PD. Further research should be conducted to establish whether TQ represents a promising therapeutic drug with which to modulate the Nrf2-ARE signaling pathways to confer strong neuroprotective effects against the development of PD.

In the present study, we established a MPP^+^-induced cytotoxicity model, and a mouse model of 1-methyl-4-phenyl-1, 2, 3, 6-tetrahydropyridine (MPTP)-induced PD, to investigate the biomechanisms that may underlie the neuroprotective effects of TQ. Our findings suggested that TQ activates the Nrf2 signaling cascade in PD and therefore represents a potential drug candidate for the treatment of PD.

## Materials and Methods

### Raw Materials and Reagents

We purchased TQ (99% pure), MPP^+^ (≥98% pure), MPTP (≥98% pure), and 3-(4, 5-dimethylthiazol-2-yl)-2, 5-diphenyltetrazolium bromide (MTT) from Sigma (St. Louis, United States). Modified Eagle’s medium (MEM), F12, fetal bovine serum, dimethyl sulfoxide (DMSO), and trypsin-EDTA were purchased from Gibco BRL (Grand Island, NY). Reactive oxygen species (ROS) assay kits and a nuclear and cytoplasmic protein extraction kit from Shanghai YESEN Biotech Co., Ltd. (Shanghai, China). We also purchased an 8-hydroxy-deoxyguanosine (8-OHdG) ELISA kit from Cusabio (Houston, United States). Antibodies against BAX (ab32503), Bcl-2 (ab196495), caspase 3 (ab4051), Nrf 2 (ab137550), HO-1 (ab13243), NQO1 (ab34173), GST (ab111947), tyrosine hydroxylase (TH) (ab137869), α-synuclein (ab212184), β-actin (ab8227), and Lamin B1 (ab16048) were purchased from Abcam (London, United Kingdom). Nitrocellulose membrane was purchased from GE Healthcare Life Sciences (Pittsburgh, PA, United States). Radio Immunoprecipitation Assay (RIPA), phenylmethylsulfonyl fluoride (PMSF), a bicinchoninic acid (BCA) protein quantification kit, SOD assay kits, an MDA assay kit, and a glutathione peroxidase (GSH-Px) kit were purchased from Beijing Solarbio Science & Technology Co., Ltd. (Beijing, China). We also purchased a NucleoSpin RNAII isolation kit and a cDNA RT kit from Tiangen Science and Technology Co., Ltd. (Beijing, China). Lipofectamine 3,000 reagent was purchased from Hanbio Biotechnology Co., Ltd.

### Cell Growth and Treatment

Human neuroblastoma SH-SY5Y cells were obtained from a cell bank held by the Chinese Academy of Sciences and were cultured in a 1:1 mixture of minimum Eagle’s medium (MEM ) and F12 containing 100 IU/ml of penicillin, 0.1 mg/ml of streptomycin, and 10% heat-inactivated fetal calf serum. Cells were cultured in a humidified atmosphere with 5% CO_2_ at a temperature of 37°C. Neuronal differentiation was triggered by adding 10 μM retinoic acid (RA) in MEM/F12 containing 10% FBS and allowing cells to culture for a further 7 days. A 10 mM stock solution of TQ was prepared in DMSO. We ensured that the final concentration of TQ added to MEM/F12 did not exceed 0.01%. We performed siRNA interference by transfecting cells with Nrf2 siRNA or negative control (NC) siRNA in a 6-well plate using Lipofectamine 3,000 reagent for 24 h in accordance with the manufacturer’s guidelines. After 24 h of transfection, the cells were treated with MPP^+^ with or without TQ. The cells were then separated into seven groups: 1) a control group in which RA-differentiated SH-SY5Y cells were administered with MEM/F12 containing 10% fetal calf serum; 2) a model group in which RA-differentiated SH-SY5Y cells were administered with 1 mM MPP^+^ for 24 h; 3) a TQ-treated group in which RA-differentiated SH-SY5Y cells were pre-treated with TQ (0.25, 0.5, and 0.75 μM) for 2 h and then exposed to 1 mM MPP^+^ for 24 h; 4) a negative control (NC) siRNA group in which RA-differentiated SH-SY5Y cells were administered with NC siRNA for 24 h followed by 1 mM MPP^+^ for 24 h; 5) a TQ-treated NC siRNA group in which RA-differentiated SH-SY5Y cells were administered with NC siRNA for 24 h followed by TQ with MPP^+^; 6) a Nrf2 siRNA group in which RA-differentiated SH-SY5Y cells were treated with Nrf2 siRNA for 24 h followed by 1 mM MPP^+^ for 24 h; and 7) a TQ-treated Nrf2 siRNA group in which RA-differentiated SH-SY5Y cells were administered with Nrf2 siRNA for 24 h followed by TQ with MPP^+^.

### Animals and Drug Administration

All *in vivo* experiments involved C57/BL6 mice (Beijing Vital River Laboratory Animal Technologies Co. Ltd, Beijing, China). All mice (age: 4–5 months; weight: 25–30 g) were housed in standard laboratory cages (three to five mice per cage) under typical laboratory conditions (12-h light/dark cycle, a temperature of 20–22°C, and a humidity of 50–60%) and were provided with food and water *ad libitum*. The mice were handled in strict accordance with guidelines provided by the National Institutes of Health Guide for the Care and Use of Laboratory Animals. For gene knockdown, lentivirus-wrapped negative control siRNA (NC), lentivirus-wrapped Nrf2 siRNA with GFP (siNrf2-GFP) or lentivirus-wrapped Nrf2 siRNA (siNrf2; GenePharma, Shanghai, China) were injected into the tail vein (20 μl/mouse, 10^9^ TU/ml). After 1 week, mice received a daily intraperitoneal (i.p.) injection of 25 mg/kg MPTP for 5 days. For the TQ treatment studies, mice received a daily injection (i.p) of 10 mg/kg body weight TQ or normal saline for 1 week, starting on the day before each dose of MPTP. At the end of TQ or saline treatment, the mice were culled, and their brains were harvested for analysis. We randomly divided the mice into eight groups: a control group receiving only the vehicle; a TQ group receiving TQ treatment; an MPTP group to act as a model for PD; an MPTP + TQ group in which the PD model was treated with TQ; an NC group in which the PD model was injected with negative control siRNA; an NC + TQ group in which the PD model was injected with NC siRNA and TQ; an siNrf2 group in which the PD model was injected with Nrf2 siRNA; and an siNrf2+TQ group in which the PD model was injected with Nrf2 siRNA and TQ.

### Assessment of Cell Viability

We seeded SH-SY5Y cells (2 × 10^4^ cells/well) into 96-well culture plates. Following adherence, we pre-treated SH-SY5Y cells with TQ (0.25, 0.5, and 0.75 μM) for 2 h followed by exposure to 1 mM MPP^+^ for 24 h. At the end of the experiment, we used the MTT assay to investigate cell viability.

### Biochemical Examination

SH-SY5Y cells were incubated in culture medium containing TQ (0.25, 0.5, and 0.75 μM) for 2 h and treated with MPP^+^ for 24 h. The contents of MDA and the activity of SOD and GSH-Px were measured in accordance with the manufacturer’s guidelines. Briefly, cells and brain tissues were homogenized, sonicated, and centrifuged. The concentration of protein in the supernatants was measured using BCA protein quantification kit. Then, we measured levels of MDA and activities of SOD and GSH-Px, using commercially available kits following the instructions provided by the manufacturer. The multimode microplate reader was used to determine the content of MDA and the activities of SOD and GSH-Px in the samples at 532 , 560 , and 340 nm.

### TUNEL Staining

The quantification of apoptotic neuronal cells was measured using TUNEL staining method following the instructions provided by the manufacturer and as previously described (Wu et al., 2017). The samples were visualized using an ortho-fluorescent microscope by an experienced pathologist blind to the experimental condition, and the TUNEL-positive cells were assessed.

### Estimation of Reactive Oxygen Species Production

Levels of ROS were determined in cells using a ROS assay kit containing dichlorofluorescein diacetate (DCFH-DA). SH-SY5Y cells were grown in 6-well plates treated with TQ and/or MPP^+^. On the day of the experiment, we removed the medium and incubated the cells with 10 μM DCFH-DA for 20 min at 37°C. We then used PBS to wash off excess DCFH-DA and then determined the relative levels of fluorescence by flow cytometry at an excitation wavelength of 480 nm and an emission wavelength of 525 nm.

### 8-OHdG Evaluation

Brain tissues were removed from culled mice and ground gently in an automatic grinder for 1 min at room temperature. Next, each sample was diluted by 5-fold and the concentration of 8-OHdG was determined by Enzyme-linked immunosorbent assay (ELISA) in accordance with the manufacturer’s guidelines. Absorbance was measured at a wavelength of 450 nm on a microplate reader (BioTeK, United States).

### RT-qPCR

RNA was isolated from SH-SY5Y cells using the NucleoSpin RNAII isolation kit (Tiangen, China) in accordance with the manufacturer’s guidelines. RNA was then reverse transcribed into cDNA using a High Capacity cDNA RT kit (Qiagen, Hilden, Germany). Real-time polymerase chain reaction (RT-PCR) was then performed using primers designed to *Nrf2* (forward,5’-GCATTCTCTAACTTGTTTGGTGGG-3’; reverse, 5’-CAA​TAG​CCC​AGG​TAG​CCA​CTC​A-3’) and *β-actin* (forward, 5’-GTG​ACG​TTG​ACA​TCC​GTA​AAG​A-3’; reverse, 5’-GTA​ACA​GTC​CGC​CTA​GAA​GCA​C-3’). PCR was performed on a StepOne Plus real-time PCR system (ABI, Darmstadt, Germany) using 2.5 µl of cDNA template, 2 μl of each primer (7.5 μM), 12.5 μl of FastStar universal SYBR Green Master, and 8 μl of ddH_2_O per sample. The following cycling conditions were used: an initial step at 95°C for 10 min, followed by 40 cycles of 95°C for 15 s, 60°C for 60 s, and 60–95°C, 0.3 °C/15 s. *β-actin* was used as a reference gene. Data analysis is based on the ΔΔCt method and the raw data is normalized to *β*-actin.

### Immunofluorescence and Immunohistochemistry

Immunofluorescence (IF) and immunohistochemical (IHC) staining were performed as described previously (Han et al., 2020). In brief, mouse brains were perfused transcardially with 4% paraformaldehyde (PFA) and then fixed in PFA overnight. Brain tissues were then sectioned through the region of interest at a thickness of 20–30 μm. Antigen retrieval was then carried out by incubating sections in sodium citrate at pH 6.0 for 15–30 min at 80°C. The sections were then rinsed three times with 1X PBS and endogenous peroxidase was blocked using 3% H_2_O_2_ at room temperature for 15 min. The sections were then blocked with 5% BSA in 0.3% Triton X-100 in PBS for 1 h at RT. Next, we incubated the sections overnight with an anti-TH antibody (1:1,000, Abcam, United Kingdom) and an anti-α-synuclein antibody (1:1,000, Abcam) at 4°C. The following morning, the sections were washed three times in PBS and then incubated with a secondary antibody for 1 h at room temperature. Positive immunoreactivity was then developed using DAB and examined with an Olympus BX53 microscope (Olympus, Tokyo, Japan).

For fluorescent staining, the sections were blocked with 3% BSA for 30 min and then incubated overnight with anti-TH antibody (1:250; Abcam) and anti-α-synuclein antibody (1:250; Abcam, United Kingdom) at 4°C. The following morning, the sections were rinsed three times in PBS and then incubated with fluorescent secondary antibodies for 1 h in the dark at room temperature. Subsequently, the sections were rinsed in PBS, counterstained with DAPI (Life Technologies) and mounted using Prolong Gold anti-fade (Servicebio, China). The samples were then evaluated using an Olympus BX53 microscope and images were analyzed using ImageJ (NIH, Bethesda, MD, United States).

### Fluoro-Jade B Staining

The brain slides were incubated with FJB working solution (0.1% acetic acid) at 4°C overnight, rinsed with distilled water, and dried in an oven at 50–60°C for 15 min. Finally, the sections were visualized, and the number of FJB-positive cells was counted under BX53 microscope.

### Western Blot Analysis

The nuclear protein was extracted and separated according to the nuclear and cytoplasmic protein extraction kit. SH-SY5Y cells were grown to 70–80% confluency in a culture flask and then cultured at 37°C in 5% CO_2_ with TQ and/or MPP^+^ for 24 h. Subsequently, cells were washed in PBS and lyzed with cytoplasmic protein extraction agent for 15 min. The samples were then centrifuged for 10 min at 700×g at 4°C and the supernatants were collected for further analysis. Then the nuclear protein extraction agent was added in the pellet. After 15 times vortex for 30 min and 14,000×g centrifuging for 10 min at 4°C, the supernatants were obtained as nuclear extracts. We also harvested a striatum tissue lysate by treating the lysate buffer with proteinase and phosphatase inhibitors. BCA was used to quantify the concentrations of protein samples acquired from each experimental group. For each sample, we separated 30 µg of protein per lane on a denaturing sodium dodecyl sulfate polyacrylamide gel electrophoresis (SDS-PAGE) gels and transferred the separated proteins onto nitrocellulose filter membranes. Next, membranes were blocked with non-fat dried milk for 1 h at room temperature and incubated overnight at 4°C with primary antibodies against Bax (1:1,000), Bcl-2 (1:1,000), caspase-3 (1:1,000), Nrf-2 (1:1,000), HO-1 (1:2000), NQO1 (1:1,000), GST (1:1,000), TH (1:5,000), α-synuclein (1:1,000), β-actin (1:1,000), and Lamin B1 (1:500). The following morning, membranes were washed and incubated with appropriate secondary antibodies (1:1,000). Positive signals were then visualized by ECL (Thermo, United States) and imaged using an Image Quant chemiluminescence system (Tanon, China). Densitometric analysis was performed using ImageJ.

### Statistical Analysis

Graph Pad Prism 8 (GraphPad, La Jolla, CA, United States) was used for all data analysis and to prepare graphs. Data are presented as the mean ± standard error of the mean (SEM). The Student’s *t*-test was used to analyze single variables. One-way or two-way analysis of variance (ANOVA) was used to compare differences between the means of multiple groups. *p ≤* 0.001 signified high significance (***), *p ≤* 0.01 signified moderate significance (**), *p ≤* 0.05 signified significant differences (*), and *p* > 0.05 indicated non-significant differences.

## Results

### Thymoquinone Prevented MPP^+^-Induced Cell Death in SH-SY5Y Cells

**FIGURE 1:**
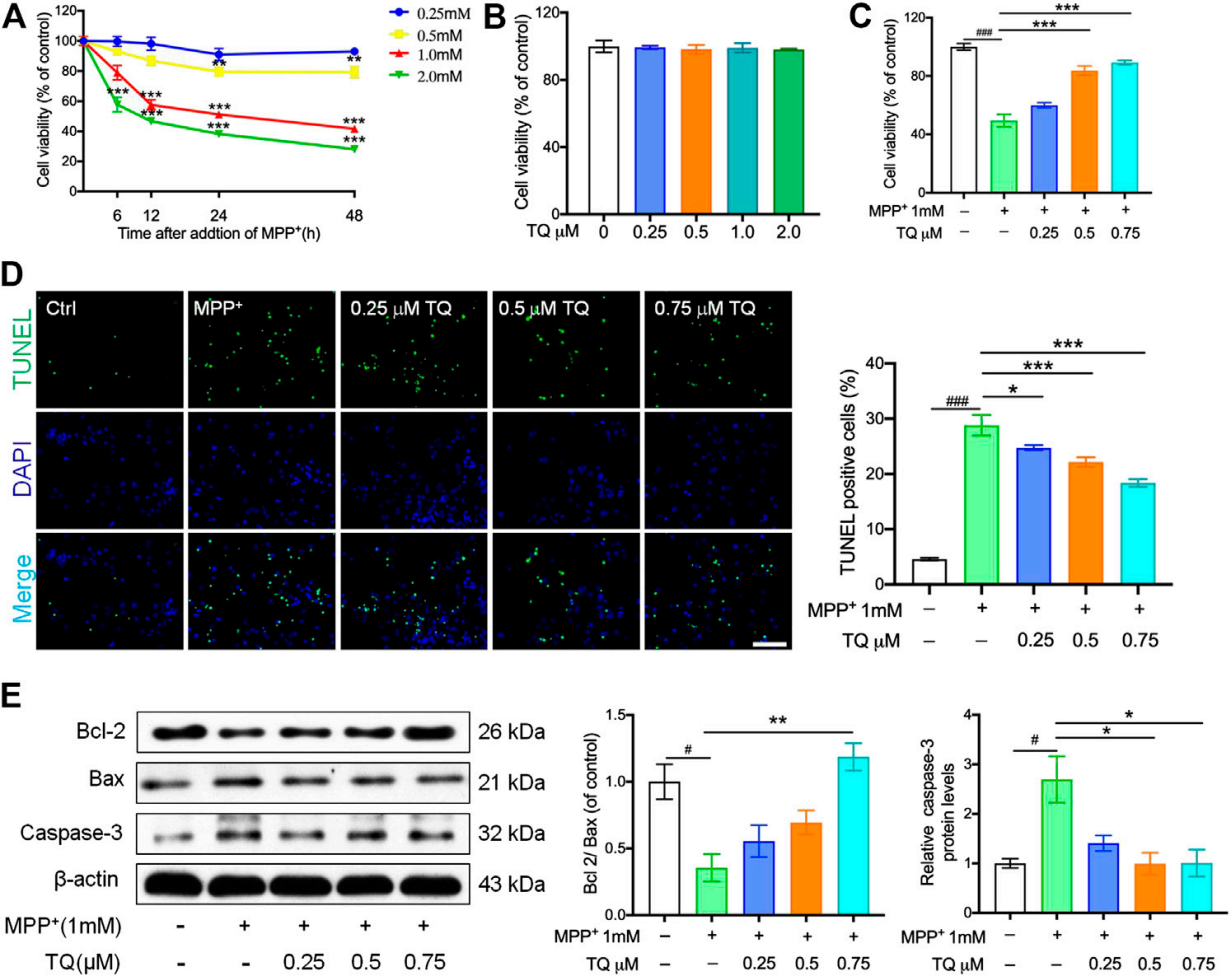
TQ prevented MPP^+^-induced cell death in SH-SY5Y cells. **(A)** SH-SY5Y cells were treated with MPP^+^ (0.25, 0.5, 1.0, and 2.0 mM) for 6, 12, 24, and 48 h, cell viability were measured by MTT assays (*n* = 6). **(B)** Cell viability of SH-SY5Y cells in different TQ group for were measured at 24 h (*n* = 6). **(C)** Pretreated MPP^+^-induced SH-SY5Y cells with TQ (0.25, 0.5, and 0.75 μM), cell viability were measured by MTT assays (*n* = 6). **(D)** Terminal deoxynucleotidyl transferase dUTP nick end labeling (TUNEL) staining. Sections were labeled by TUNEL (green) to detect apoptotic cells and counterstained with DAPI (blue) to detect nuclei. Scale bar, 100 μm. **(E)** Bcl2/Bax ratio and the levels of caspase-3 in MPP^+^-induced SH-SY5Y cells with or without TQ pretreatment were quantified by western blotting (*n* = 3). Data are indicated as the mean ± SEM; one-way ANOVA, ^#^ *p* < 0.05, ^##^ *p* < 0.01,^###^ *p* < 0.001, relative to the control group; ^*^ *p* < 0.05, ^*^^*^ *p* < 0.01, ^*^^*^^*^ *p* < 0.001, relative to the MPP^+^ group.

SH-SY5Y cells were administered with a series of MPP^+^ concentrations over a known time period. Our analyses showed that MPP^+^ reduced cell viability in a time- and concentration-dependent manner. Relative to the control group, SH-SY5Y cells showed significantly reduced levels of viability (to 51.2 ± 4.9%) when treated with 1 mM MPP^+^ for 24 h. Hence, SH-SY5Y cells were treated with 1 mM MPP^+^ for 24 h to create an injury model for PD in all subsequent experiments (Figure 1A). Next, we investigated the impact of TQ on cell viability. When administered at concentrations of 0.25–2.0 μM for 24 h, TQ treatment had no cytotoxic effects on SH-SY5Y cells (Figure 1B). Next, we investigated the neuroprotective effects of TQ in SH-SY5Y cells. Treatment with 1 mM MPP^+^ significantly reduced the viability of SH-SY5Y cells while different concentrations of TQ pre-treatment (0.25, 0.5, and 0.75 μM) efficiently blocked MPP^+^-induced cell death (Figure 1C).

Compared to the control group, the numbers of TUNEL-positive cells in the MPP^+^ group were significantly elevated; however, TQ treatment caused a significant reduction in the number of TUNEL-positive cells (Figure 1D). In addition, we used western blotting to determine the levels of caspase-3 in SH-SY5Y cells; this is a standard technique that is commonly used to assess apoptosis. We found that the expression levels of Bax were upregulated after treatment with MPP^+^ for 24 h while the levels of Bcl-2 were downregulated. Pretreatment with TQ suppressed the increase levels of Bax expression in SH-SY5Y cells and suppressed the reduction in Bcl-2 expression (Figure 1E). Similarly, TQ treatment reduced the MPP^+^-mediated increase in caspase-3 expression, thus suggesting that TQ inhibited MPP^+^-mediated apoptosis (Figure 1E).

### Thymoquinone Suppressed MPP^+^-Induced Oxidative Stress and Activated the Nrf2-ARE Signaling in Pathway SH-SY5Y Cells

**FIGURE 2:**
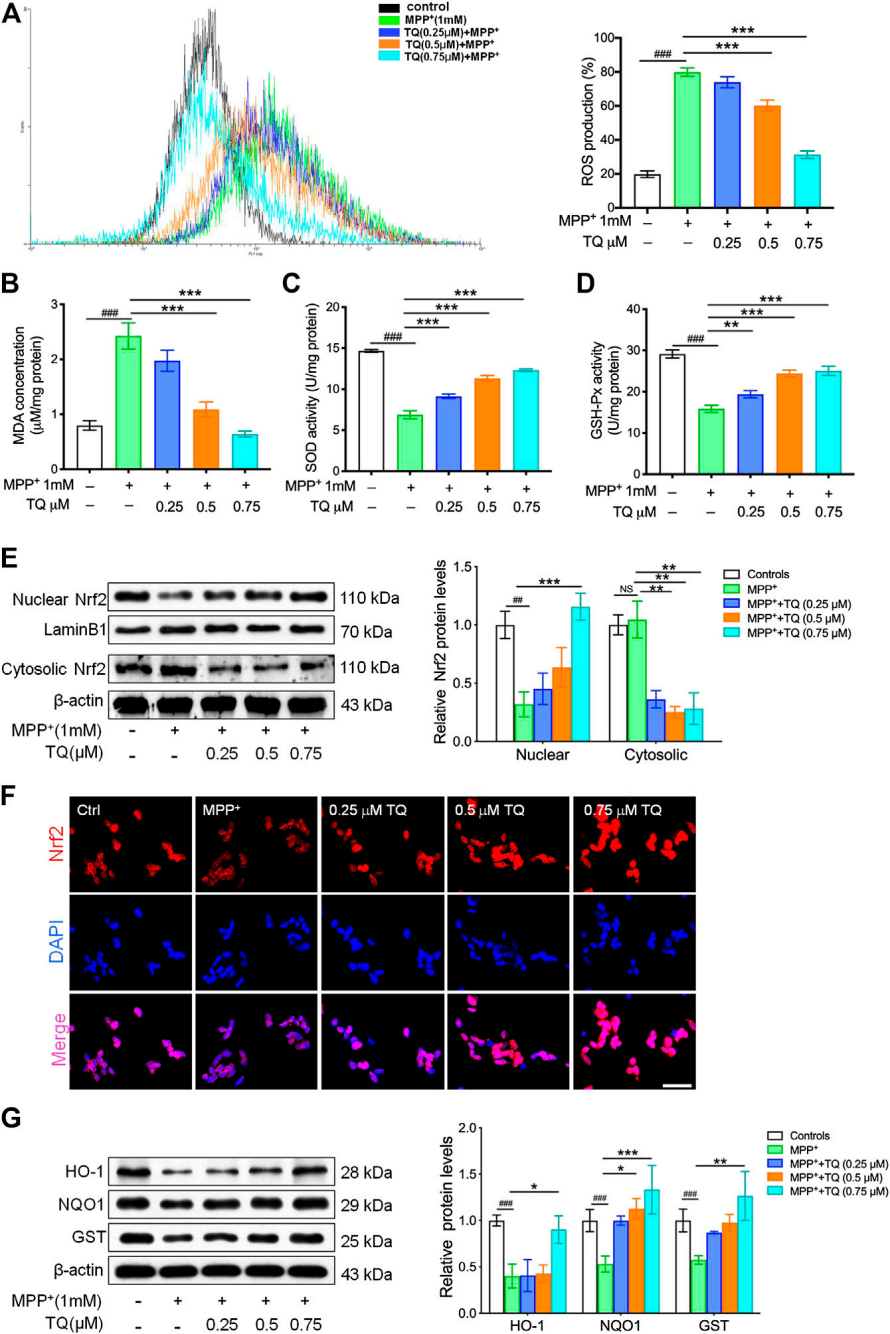
TQ inhibited MPP^+^-induced oxidative stress in SH-SY5Y cells. **(A)** Levels of ROS in MPP^+^-induced SH-SY5Y cells with or without TQ pretreatment (*n* = 6). **(B)** Levels of MDA in MPP^+^-induced SH-SY5Y cells with or without TQ pretreatment (*n* = 6). **(C)** The activities of SOD in MPP^+^-induced SH-SY5Y cells with or without TQ pretreatment (*n* = 6). **(D)** The activities of GSH-Px in MPP^+^-induced SH-SY5Y cells with or without TQ pretreatment (*n* = 6). **(E)** Western blot analysis of nuclear translocation of Nrf2 indicated by TQ treatment when exposured to MPP^+^ in SH-SY5Y cells (*n* = 3). **(F)** Nrf2 translocation was detected by immunofluorescence. Scale bar, 100 μm. **(G)** Levels of HO-1, NQO1, and GST in MPP^+^-induced SH-SY5Y cells with or without TQ pretreatment were quantified by western blotting (*n* = 3). Data are indicated as the mean ± SEM; one-way ANOVA, ^#^ *p* < 0.05, ^###^ *p* < 0.001, relative to the control group; ^*^ *p* < 0.05, ^*^^*^ *p* < 0.01, ^*^^*^^*^ *p* < 0.001, relative to the MPP^+^ group.

Considering the balance between oxidation and antioxidation in a normal healthy cell, we next evaluated the effect of TQ on ROS generation and determined the levels of MDA and the activities of SOD and GSH-Px. A H2DCF-DA probe was used to determine the specific rate of ROS generation. MPP^+^ increased the rate of ROS production; however, TQ treatment significantly reduced this increase in ROS generation (Figure 2A). Following treatment with 1 mM MPP^+^, there was a significant rise in MDA levels relative to the control group; furthermore, TQ reduced MDA activity levels in a concentration-dependent manner (Figure 2B). In the model group, the bioactivities of certain antioxidant enzymes (SOD and GSH-Px) were significantly reduced. However, TQ treatment increased the activities of SOD and GSH-Px in MPP^+^-induced SH-SY5Y cells (Figures 2C,D). We next examined the effect of TQ on the nuclear translocation of Nrf2. Relative to the model group, the expression of nuclear Nrf2 was markedly upregulated when treated with 0.5 and 0.75 μM of TQ (Figure 2E). Furthermore, the result of immunofluorescence staining showed that TQ-treatment led to increasing the level of nuclear translocation of Nrf2 in MPP^+^-induced SH-SY5Y cells (Figure 2F). These observations suggested that TQ promotes Nrf2 nuclear translocation. Following the nuclear translocation of Nrf2, we also investigated the expression of several proteins downstream of the Nrf2-ARE cascade (HO-1, NQO1, and GST) by western blotting following the pretreatment of MPP^+^-induced SH-SY5Y cells with TQ. We found that the levels of these proteins were significantly upregulated in SH-SY5Y cells that were pre-treated with TQ (Figure 2G). These data demonstrate that TQ might attenuate oxidative stress caused by MPP^+^ in SH-SY5Y cells.

### Thymoquinone Suppressed MPP^+^-Induced Cytotoxicity in SH-SY5Y Cells in a Manner That Was Dependent on Nrf2

**FIGURE 3:**
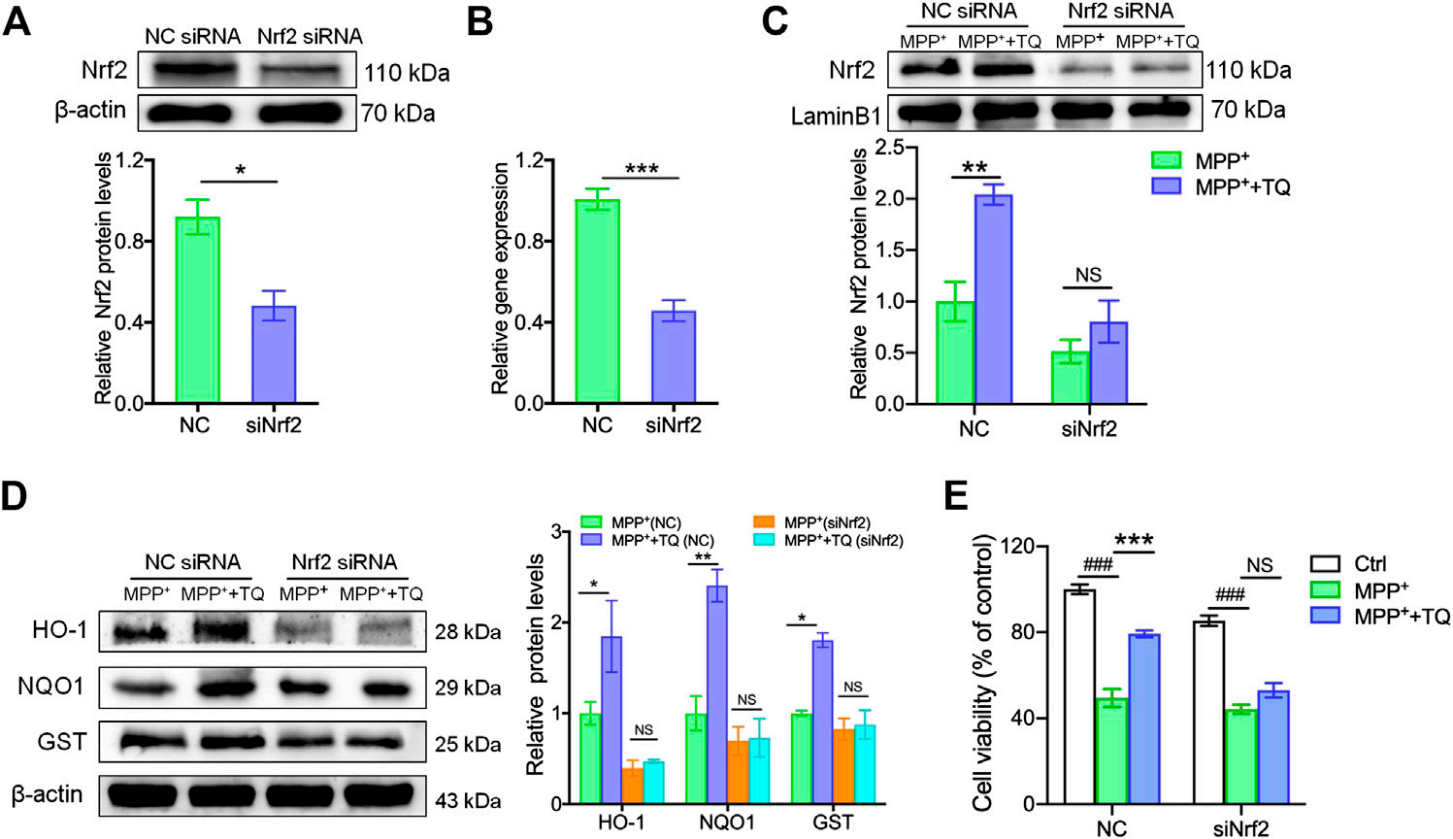
The Nrf2/ARE pathway was involved in the antioxidative effects induced by TQ in SH-SY5Y cells. **(A,B)** The protein and mRNA levels of Nrf2 in SH-SY5Y cells transfected with Nrf2 siRNA or NC siRNA (*n* = 3). **(C)** Levels of Nrf2 in SH-SY5Y cells transfected with Nrf2 siRNA or NC siRNA with or without TQ pretreatment were measured by western blotting (*n* = 3). **(D)** Levels of HO-1, NQO1, and GST in SH-SY5Y cells transfected with Nrf2 siRNA or NC siRNA with or without TQ pretreatment were quantified by western blotting (*n* = 3). **(E)** The cell viability of SH-SY5Y cells transfected with Nrf2 siRNA or NC siRNA with or without TQ pretreatment (*n* = 6). Data are indicated as the mean ± SEM; Student’s *t*-test, two-way ANOVA, ^###^ *p* < 0.001, relative to the control group; ^*^ *p* < 0.05, ^*^^*^ *p* < 0.01, ^*^^*^^*^ *p* < 0.001, relative to the corresponding NC siRNA group.

To explore the potential role of the Nrf2/ARE signaling cascade in the TQ-mediated prevention of MPP^+^-induced cytotoxicity in SH-SY5Y cells, we investigated the suppressive influence of TQ on MPP^+^-induced neurotoxicity in SH-SY5Y cells. We did this by transfecting cells with either NC siRNA or Nrf2 siRNA in order to elucidate whether the Nrf2-dependent cascade was responsible for the neuroprotective effects of TQ against MPP^+^-induced oxidative apoptosis. Western blotting and RT-PCR results indicated that Nrf2 siRNA successfully silenced the expression of Nrf2 in cells while the NC siRNA did not (Figures 3A,B). The silencing of Nrf2 in SH-SY5Y cells markedly reduced the expression of Nrf2. The expression of nuclear Nrf2 was significantly elevated after pretreating SH-SY5Y cells transfected with NC siRNA with TQ. Nevertheless, Nrf2 silencing suppressed the increased nuclear translocation of Nrf2 induced by TQ in SH-SY5Y cells (Figure 3C). Moreover, the expression of several proteins downstream of the Nrf2-ARE axis (HO-1, NQO1, and GST) was abolished in Nrf2-silenced SH-SY5Y cells. The expression levels of these proteins were not affected in the group of cells transfected with Nrf2 siRNA and pre-treated with TQ (Figure 3D). Furthermore, the TQ-induced increase cell viability was repressed in Nrf2-silenced SH-SY5Y cells, as determined by MTT analysis. Relative to the cells transfected with NC siRNA, the silencing of Nrf2 in SH-SY5Y cells resulted in increased susceptibility to MPP^+^ cytotoxicity (Figure 3E). These results illustrated that the cytoprotective effects of TQ on MPP^+^-treated SH-SY5Y cells were mediated through an Nrf2-dependent pathway.

### Thymoquinone Provided Biological Protection Against Nigrostriatal Dopaminergic Degeneration in an Experimental Model of Parkinson’s disease

**FIGURE 4:**
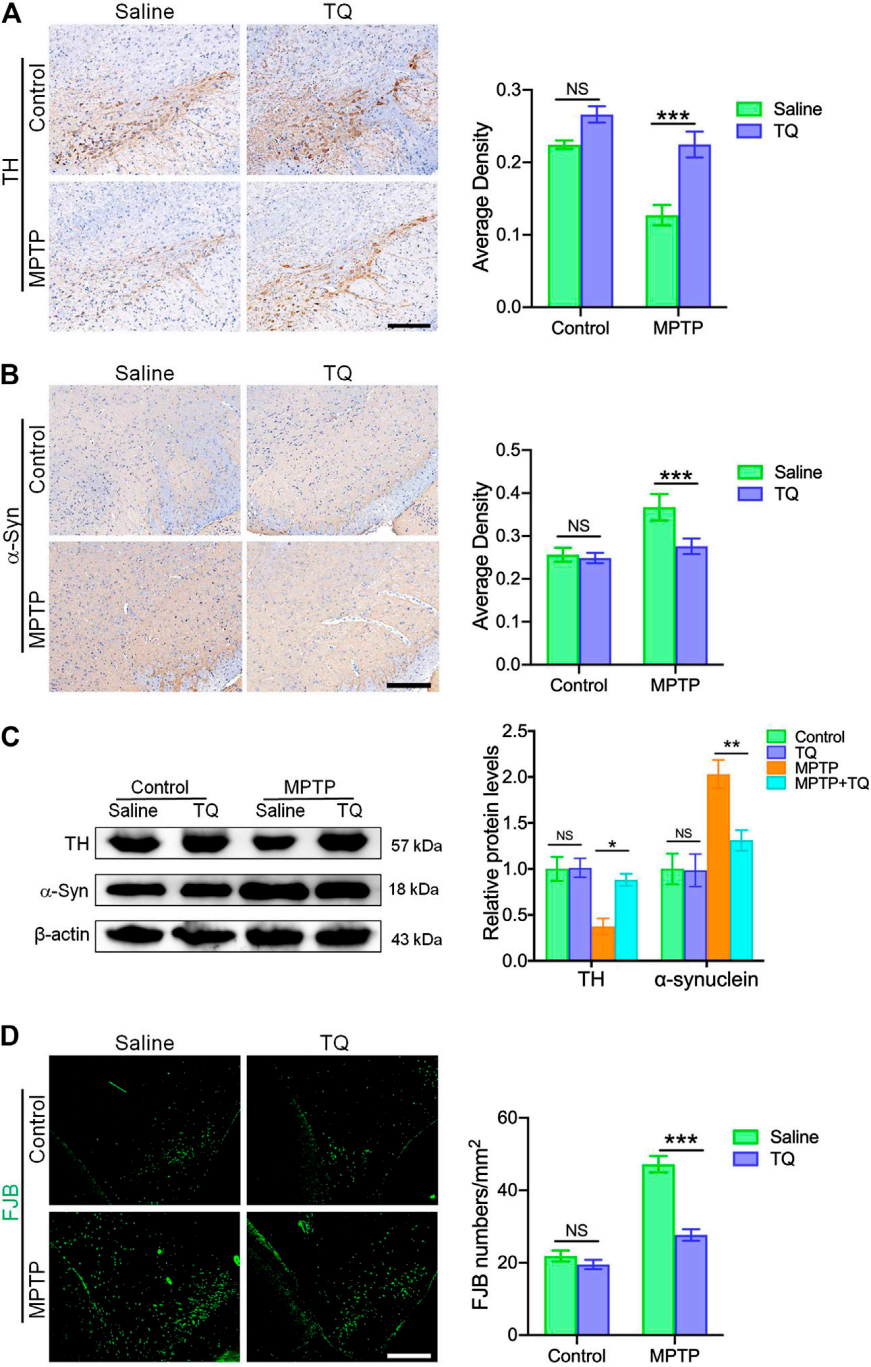
TQ treatment provided bioprotective effects against nigrostriatal dopaminergic degeneration in the MPTP model of PD. **(A)** Immunohistochemical staining and quantification of TH^+^ SNc dopaminergic neurons in TQ or saline-treated MPTP mice (*n* = 4). Scale bar, 100 μm. **(B)** Immunohistochemical staining and quantification of α-synuclein in the SNc of TQ or saline-treated MPTP mice (*n* = 4). Scale bar, 100 μm. **(C)** Levels of TH and α-synuclein in the SNc of TQ or saline-treated MPTP mice were measured by western blotting (*n* = 3). **(D)** Fluorescence imaging of FJB staining (green) in the SNc of TQ or saline-treated MPTP mice. Scale bar, 100 μm. Data are indicated as the mean ± SEM; two-way ANOVA, ^*^ *p* < 0.05, ^*^^*^ *p* < 0.01, ^*^^*^^*^ *p* < 0.001, relative to the corresponding saline group.

In order to investigate the therapeutic effects of TQ in PD, we investigated the expression of TH and α-synuclein in the substantia nigra pars compacta (SNc) of an MPTP mouse model using IHC staining. Following MPTP treatment, we observed a significant depletion of TH^+^ dopaminergic neurons in the SNc when compared with the control group. However, TQ treatment reduced the depletion of TH^+^ dopaminergic neurons in the SNc (Figure 4A). Previous research has shown that high levels of α-synuclein represent an important pathological characteristic of PD (Rocha et al., 2018). Therefore, to confirm that TQ facilitated the repression of α-synuclein-induced neurodegeneration, we investigated the expression of α-synuclein by IHC staining. Analysis revealed that there was an increase in α-synuclein immunoreactivity in mice administered with MPTP when compared with the control group. However, TQ treatment remarkably reduced the expression of α-synuclein in the SNc when compared to MPTP alone (Figure 4B). Similarly, western blotting assays of SNc lysates demonstrated that the expression of TH protein was reduced in the MPTP group, although this trend was attenuated by TQ treatment (Figure 4C). Consistent with IHC findings in the SNc, the expression of α-synuclein was significantly elevated in the SNc of mice following MPTP injection. However, TQ treatment reduced the levels of α-synuclein in the SNc (Figure 4C). We also investigated the neuroprotective effects of TQ using FJB staining which identifies denatured neurons by emitting green fluorescence. This assay showed that exposure to MPTP was accompanied by neurodegeneration in the mouse model of PD and that these effects were alleviated by TQ treatment (Figure 4D). Therefore, our *in vivo* work indicated that TQ provides protection for nigrostriatal dopaminergic neurons in the mouse model of PD model against MPTP neurotoxicity.

### Thymoquinone Attenuated Oxidative Stress and Activated the Nrf2-ARE Pathway in MPTP-Treated Mice

**FIGURE 5:**
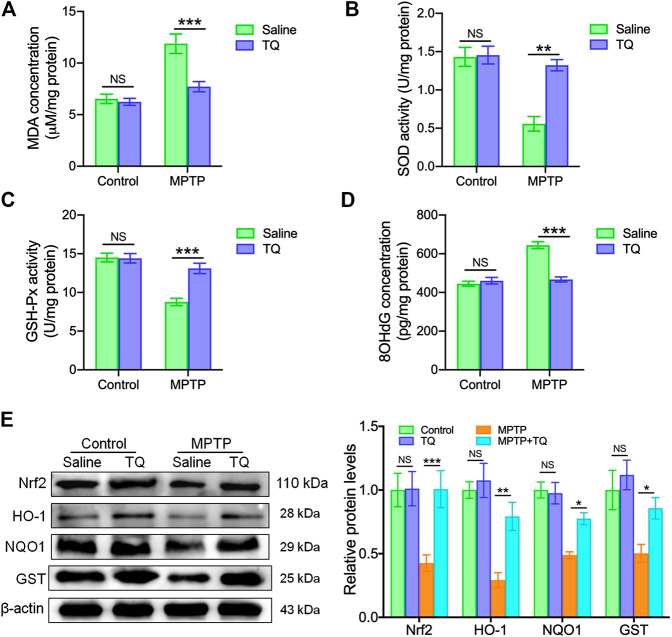
TQ abolished oxidative stress and activated the Nrf2-ARE cascade *in vivo*. **(A)** Levels of MDA in the SNc of TQ or saline-treated MPTP mice (*n* = 6). **(B,C)** The activities of antioxidant enzymes (SOD and GSH-Px) in the SNc of TQ or saline-treated MPTP mice (*n* = 6). **(D)** Levels of 8-OHdG in the SNc of TQ or saline-treated MPTP mice (*n* = 6). **(E)** Levels of Nrf2, HO-1, NQO1, and GST in the SNc of TQ or saline-treated MPTP mice were quantified by western blotting (*n* = 3). Data are indicated as the mean ± SEM; two-way ANOVA, ^*^ *p* < 0.05, ^*^^*^ *p* < 0.01, ^*^^*^^*^ *p* < 0.001, relative to the corresponding saline group.

Next, we investigated whether TQ provided bioprotective effects for dopaminergic neurons against MPTP neurotoxicity *in vivo* and whether this was mediated via its antioxidant properties. First, we investigated oxidative activity in the SNc tissue of mice with PD. We found that the levels of MDA, a crucial product of membrane lipid oxidation, were significantly elevated in the SNc following MPTP exposure but were suppressed by TQ treatment in the SNc (Figure 5A). SOD and GSH-Px are antioxidant markers and have the ability to prevent damage being incurred by important cell components (Niedzielska et al., 2016). Mice treated with MPTP expressed significantly reduced activities of SOD and GSH-Px in the SNc; these effects were reversed by TQ treatment (Figures 5B,C). In contrast with the control group, MPTP treatment increased levels of 8-OHdG in the SNc of mice with PD. Following treatment with TQ, the levels of 8-OHdG were significantly reduced when compared with the MPTP group (Figure 5D). Collectively, these results indicated that TQ mitigates the oxidative damage induced by MPTP.

Next, we investigated whether the Nrf2-ARE cascade plays a role in the effects observed in our *in vivo* experiments. We found that the expression of Nrf2 in the SNc was significantly lower in the MPTP mice when compared with the control group (Figure 5E). However, TQ increased the level of Nrf2 in the SNc of TQ-treated mice in response to MPTP; these effects were not observed in mice treated with MPTP alone. Furthermore, three anti-oxidative genes targeted by Nrf2 (HO-1, NQO1, and GST) were significantly upregulated in the SNc following TQ treatment when compared with MPTP treatment (Figure 5E). Collectively, these data suggested that TQ activates the Nrf2-ARE signaling pathway in the mouse model of PD.

### Thymoquinone Prevented MPTP-Induced Dopaminergic Degeneration and Rescued the Depletion of TH^+^ Neurons in a Nrf2-dependent Manner

**FIGURE 6:**
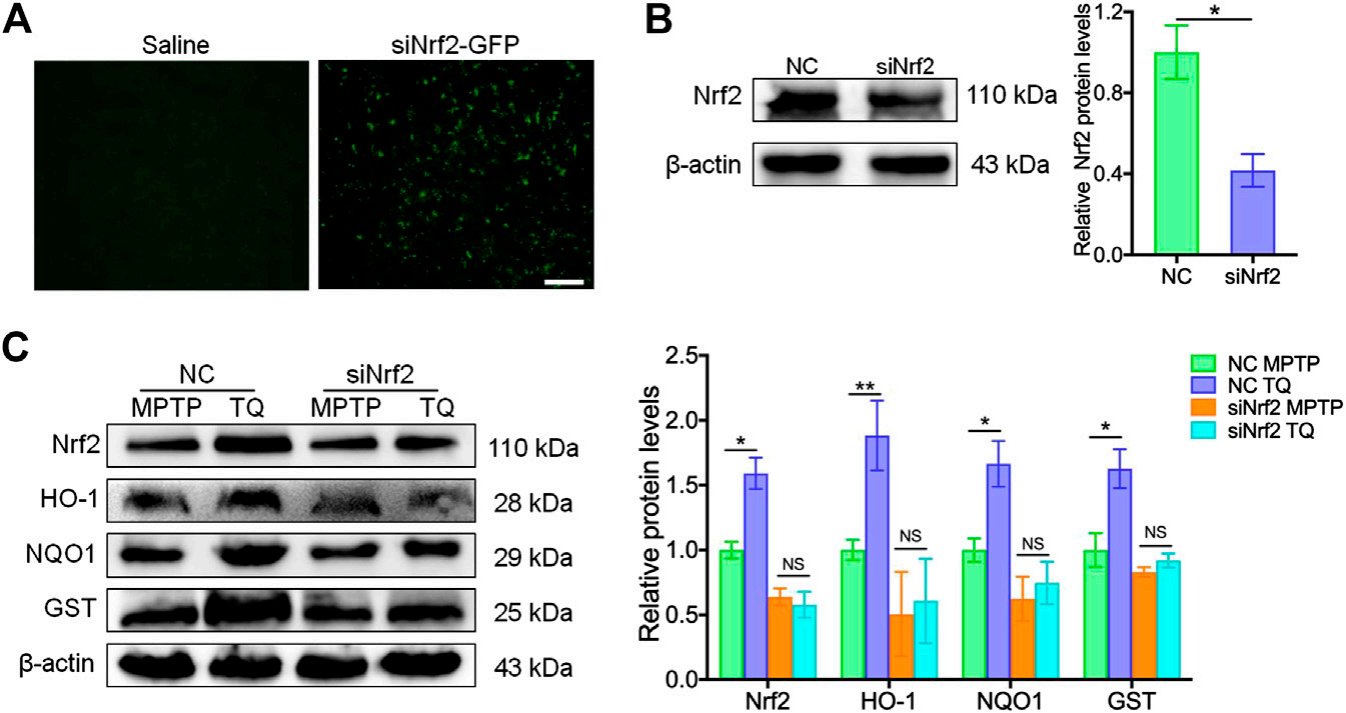
*In vivo* silencing of Nrf2 inhibited the activation of Nrf2-ARE signaling following the administration of TQ. **(A)** Immunofluorescence staining for GFP in the brain samples from lentivirus-wrapped the negative control siRNA (NC) or the Nlrp3 siRNA with the GFP (siNrf2-GFP) mice via the tail vein. Scale bars, 100 μm. **(B)** Expression of Nrf2 in brain samples from MPTP mice injected with either Nrf2 siRNA or NC siRNA (*n* = 3). **(C)** Levels of Nrf2, HO-1, NQO1, and GST in the SNc of TQ or saline-treated MPTP mice following the injection of NC or siNrf2 (*n* = 3). Data are indicated as the mean ± SEM, Student’s *T*-test, two-way ANOVA, ^*^ *p* < 0.05, ^*^^*^ *p* < 0.01, relative to the corresponding NC group.

To further determine whether Nrf2-ARE signaling plays a critical role in the neuroprotective effects of TQ in PD, we injected siNrf2 into the tails of experimental mice. After the injection of siNrf2, we observed green fluorescence in sections of brain tissue (Figure 6A) along with reduced levels of Nrf2 protein (Figure 6B). The expression of Nrf2 protein increased in the SNc of NC-injected mice following TQ treatment; furthermore, this increase was alleviated by the injection of siNrf2 (Figure 6C). Next, we determined if siNrf2 influenced the expression of genes downstream of the Nrf2-ARE pathway following TQ treatment in MPTP-treated mice. We investigated the expression of HO-1, NQO1, and GST. Following the downregulation of Nrf2 in the SNc, TQ failed to induce a significant elevation in NQO1, GST, and HO-1 expression in mice injected with siNrf2 (Figure 6C). Collectively, these data showed that the silencing of Nrf2 in the brain via *in vivo* siRNA treatment repressed activation of the Nrf2-ARE signaling following the administration of TQ in MPTP-treated mice.

**FIGURE 7:**
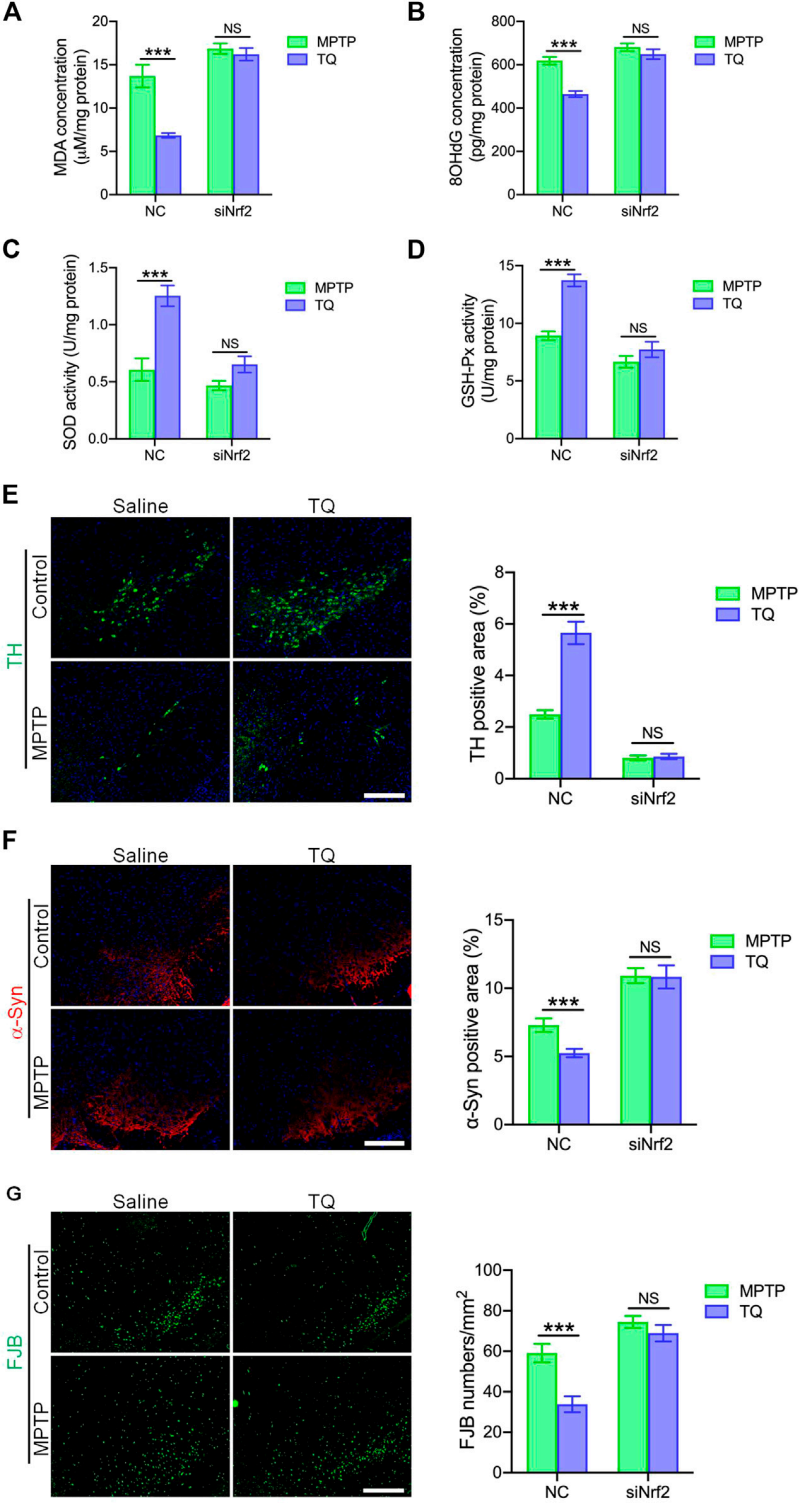
TQ prevented MPTP-induced oxidative stress and neurodegeneration in a Nrf2-dependent manner. **(A)** Levels of MDA in the SNc of TQ or saline-treated MPTP mice following the injection of either NC or siNrf2 (*n* = 6). **(B)** Levels of 8-OHdG in the SNc of TQ or saline-treated MPTP mice following the injection of either NC or siNrf2 (*n* = 6). **(C,D)** The activities of antioxidant enzymes (SOD and GSH-Px) in the SNc of TQ or saline-treated MPTP mice following the injection of either NC or siNrf2 (*n* = 6). **(E)** Immunofluorescence staining and quantification of TH (green) in the SNc of TQ or saline-treated MPTP mice following the injection of either NC or siNrf2. Scale bars, 100 μm. **(F)** Immunofluorescence staining and quantification of α-synuclein (red) in the SNc of TQ or saline-treated MPTP mice following the injection of either NC or siNrf2. Scale bars, 100 μm. **(G)** Fluorescence imaging of FJB staining (green) in the SNc of TQ or saline-treated MPTP mice following the injection of either NC or siNrf2. Scale bars, 100 μm. Data are indicated as the mean ± SEM, two-way ANOVA, ^*^ *p* < 0.001 relative to the corresponding NC siRNA group.

To investigate whether the administration of siNrf2 repressed TQ-induced antioxidation in the brain by modulating activation of the Nrf2-ARE signaling pathway, we determined the levels of two markers of oxidative stress (MDA and 8-OHdG) in MPTP-treated mice following the injection of siRNA. Our analysis identified reduced levels of MDA and 8-OHdG in the SNc of PD mice injected with NC following TQ administration. In contrast, the levels of SOD and GSH-Px showed a corresponding increase in mice injected with NC and treated with TQ. Treatment with siNrf2 reduced or even abolished the downregulation of MDA and 8-OHdG, and the upregulation of SOD and GSH-Px, following the administration of TQ (Figures 7A–D). Next, we investigated the neuroprotective effect of TQ treatment following the injection of siRNA. We observed reduced levels of TH expression and higher levels of α-synuclein in the SNc of PD mice injected with NC following the administration of TQ. However, TQ failed to alleviate MPTP-induced TH^+^ neuronal loss and the accumulation of α-synuclein in the SNc following the injection of siNrf2 (Figures 7E,F). Finally, we investigated the neuroprotective effects of TQ by FJB staining; this stain identifies denatured neurons by green fluorescence. We found that TQ treatment induced a significant reduction in the number of denatured neurons in PD mice injected with NC and that this effect was suppressed by the injection of siNrf2 (Figure 7G). Based on these results, it was evident that the anti-oxidative effects of TQ on the PD mouse model occur *via* the Nrf2 signaling cascade.

## Discussion

Globally, PD is one of the most common neurodegenerative diseases. Consequently, there is an urgent need to develop therapeutic drugs that can inhibit the neurodegenerative process associated with PD (Grayson, 2016). Presently, however, therapy is predominantly symptomatic (Beitz, 2014). Such treatment relies upon the substitution of dopamine with levodopa and is associated with a range of side effects. Oxidative stress, neuroinflammation, mitochondrial dysfunction, atypical protein aggregation, excitotoxicity, and variations in the autophagic-lysosomal cascade, are all known to be essential factors in the development and progression of PD and could be considered as intervention targets for PD therapy (Tarakad and Jankovic, 2017). Antioxidant therapy has proved very worthwhile in a range of diseases caused by ROS, including cancer, diabetes, and infectious diseases (Pennathur and Heinecke, 2004; Glasauer and Chandel, 2014). However, results from clinical trials involving the use of antioxidants to treat neurological diseases have reported conflicting outcomes with regards to their efficacy (Carvalho et al., 2017). Oxidants may play a role in PD by activating death-associated cascades and not by killing dopaminergic neurons. The Nrf2-ARE signaling cascade regulates the transcriptional expression of oxidative stress factors to re-establish redox homeostasis and is a flexible strategy for the treatment of neurodegenerative diseases (Buendia et al., 2016). Several studies have shown that Nrf2 deficiency increases the sensitivity of dopaminergic neurons to MPTP and 6-OHDA neurotoxicity, and that Nrf2-dependent antioxidants are associated with high transcriptional activity and confer protective effects in various models of PD (Chen et al., 2009; Li et al., 2018). Moreover, the activation of Nrf2 is known to confer neuroprotective effects against 6-OHDA- and MPP^+^-induced neurotoxicity (Moreira et al., 2017; Zhu et al., 2019). These findings imply that the up-regulation of the Nrf2 cascade could be exploited to design new drugs for the treatment of PD.

TQ, a significant active component in *Nigella sativa*, is a strong antioxidant and neuroprotectant (Darakhshan et al., 2015). TQ has been reported to exert efficacious anti-oxidative and anti-inflammatory effects in a model of hippocampal neurodegeneration following chronic toluene treatment (Kanter, 2008). After head injury, TQ was shown to facilitate the healing process in neural cells in a manner that was moderated by the reduction of MDA levels in the nuclei and mitochondrial membrane of neurons (Gülşen et al., 2016). Moreover, TQ has been shown to protect primary mesencephalic cells from MPP^+^-induced dopaminergic cell death (Radad et al., 2009). Another study demonstrated that TQ provides protection against MPTP-induced PD by virtue of its antioxidant and anti-inflammatory properties (Ardah et al., 2019). However, the mechanisms by which TQ can regulate cytoprotective effects against oxidative stress *in vitro* or *in vivo* have yet to be elucidated. In the present study, we investigated the efficacy of TQ as a treatment for PD using a cellular model and a mouse model of PD to identify the neuroprotective and anti-oxidative effects of TQ. We found that the protective effects of TQ were mediated by the Nrf-ARE signaling cascade, thus providing strong evidence for the use of natural products for PD therapy.

In the current study, we found that the pretreatment of MPP^+^-induced SH-SY5Y cells with TQ resulted in a significantly reduced rate of apoptosis compared with controls. Furthermore, treatment with TQ restrained the expression of the pro-apoptotic proteins Bax and caspase-3 in MPP^+^-induced SH-SY5Y cells but increased the expression levels of Bcl-2. This implies that TQ attenuates MPP^+^-induced apoptotic cell death. In addition, TQ reduced the formation of ROS by increasing the mitochondrial membrane potential. TQ also reduced the elevated levels of MDA and reversed the reduced activity of SOD and GSH-Px to levels that were within the normal range. These effects were also confirmed in an MPTP model of PD. Our results demonstrate that TQ increased the activity of anti-oxidative enzymes, including SOD and GSH-Px, reduced the levels of MDA and 8-OHdG, alleviated the depletion of dopaminergic neurons, and promoted the nuclear translocation of Nrf2 nuclear. Collectively, these processes exerted significant neuroprotective effects in the mouse model of PD. Nrf2 modulates several critical genes that can be induced by TQ, including HO-1, NQO1, and GST. Previous studies have shown that the generation of ROS results in the increased expression of HO-1 in order to provide protection to cells by amplifying antioxidant products. NQO1 has also been demonstrated to confer antioxidant properties to protect against ROS-mediated cell damage (Tufekci et al., 2011). GST codes for a detoxifying enzyme that suppresses the activation of ROS (Tufekci et al., 2011). These genes function to counter oxidative damage within brain tissues (Tufekci et al., 2011). Therefore, TQ plays a neuroprotective role by ensuring that Nrf2 is translocated into the nucleus to transactivate its target genes, thus reducing the production of ROS and helping to maintain the balance of oxidants and antioxidants. We also investigated the role of Nrf2 in the protective effects of TQ against MPP^+^-induced cytotoxicity in SHSY5Y cells *via* the transfection of Nrf2 siRNA. The transfection of Nrf2 siRNA blocked the TQ-induced expression of Nrf2-regulated genes and elevated the susceptibility of cells to MPP^+^-induced neurotoxicity. Next, we used injections of siNrf2 to silence the expression of Nrf2 protein in the mouse model of PD. Analysis revealed that the reduction of Nrf2 in the brain repressed TQ-mediated antioxidation and suppressed the TQ-induced alleviation of the depletion in dopaminergic neurons and neurodegeneration in the SNc of the mouse model of PD. These data implied that the Nrf2 signaling cascade in the brain mediated the neuroprotective effects of TQ in the mouse model of PD.

Previous research has demonstrated that TQ exerts strong anti-oxidant bioactivity and can abolish superoxide radicals, alleviate lipid peroxidation, and regenerate antioxidant enzymes (Darakhshan et al., 2015). In the present study, we found that TQ promoted Nrf2 nuclear translocation in neuronal cells and the activation of Nrf2 signaling was directly responsible for the neuroprotective effects of TQ. Further studies should now be conducted to further elucidate these mechanisms. Our study had limitations that should be considered. For example, our data were generated by the administration of TQ prior to a neuropathological injury in a mouse model. It is now important that we carry out further studies to investigate the effect of commencing treatment at the onset of injury. These studies are needed so that we can determine whether TQ is beneficial against ongoing neuropathology as this is more pathologically relevant than the scenario described herein.

## Conclusion

**FIGURE 8:**
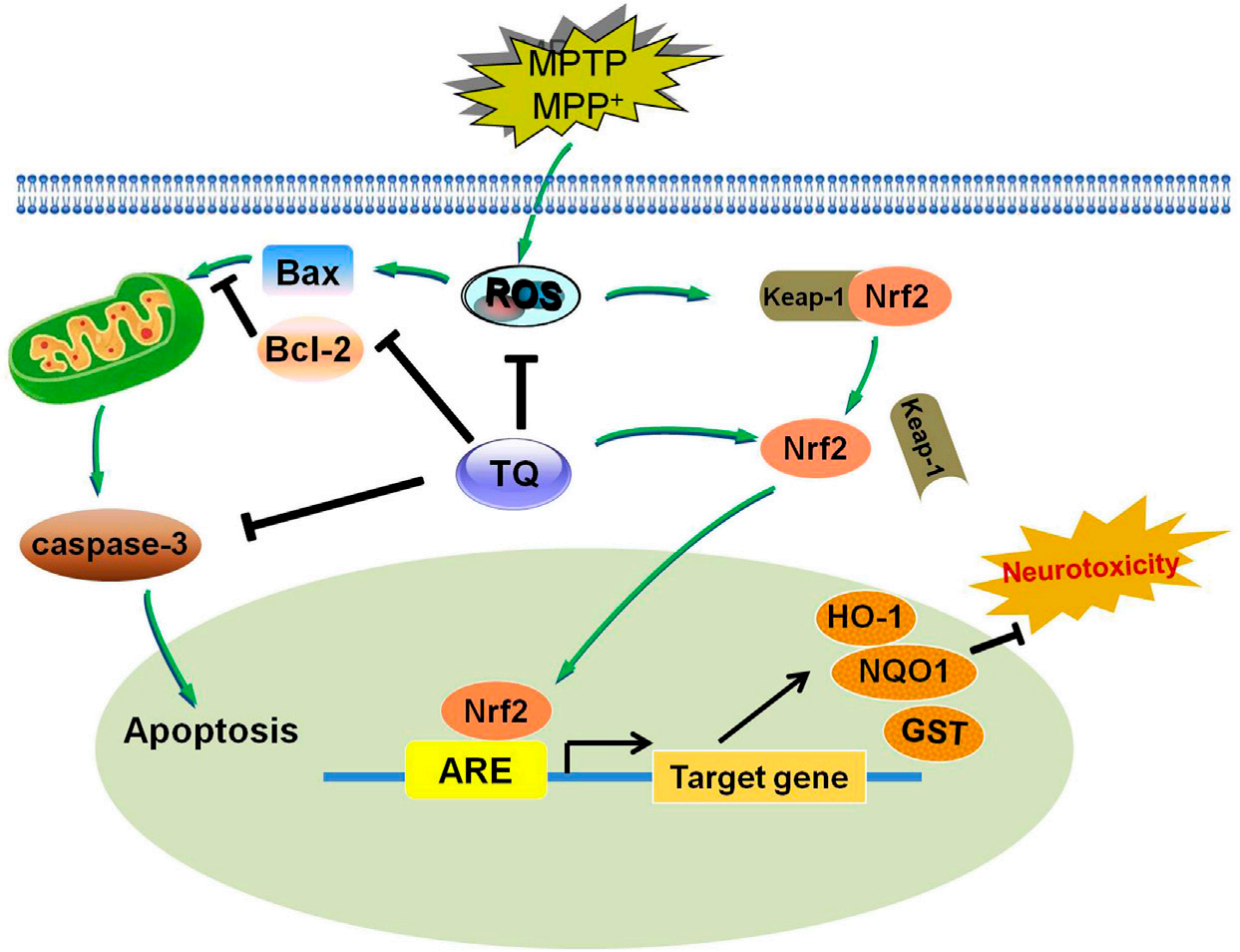
A diagram showing the proposed mechanisms of TQ resists MPTP or MPP^+^-induced oxidative stress via Nrf2/ARE pathway. TQ could inhibit MPP^+^-induced intracellular ROS formation, and inhibit MPP^+^-induced apoptosis via affecting the caspase signaling. TQ maybe stimulate the activation and nuclear translocation of Nrf2 for binding to ARE, leading to induction of HO-1, NQO1 and GST expression, thereby protecting against MPP^+^ or MPTP-induced neurotoxicity. Arrow indicates stimulation and bar indicates inhibition.

The present study found that TQ exerts neuroprotective and anti-oxidative effects on PD, both *in vivo* and *in vitro*. Our data indicated that these effects were mediated by modulation of the Nrf2-ARE signaling cascade. The administration of TQ could provide a novel therapeutic breakthrough for PD therapy (Figure 8).

## Data Availability Statement

The original contributions presented in the study are included in the article/Supplementary Material, further inquiries can be directed to the corresponding authors.

## Ethics Statement

The animal study was reviewed and approved by Hefei Institutes of Physical Science, Chinese Academy of Sciences Animal Ethics Committees (IACUC19001).

## Author Contributions

JD, NC, YH, and XW, designed the experiment. JD, XZ, SW, CX, MG, SL, and XL performed experiments. JD and SW analyzed the data and constructed the figures. JD and YH wrote and edited the manuscript, which was reviewed before submission by all authors. All authors agreed the submission of this manuscript and agreed to be accountable for all aspects of this work.

## Funding

National Natural Science Foundation of China (Nos. 81603596, 81673948, and 81774425), Natural Science Foundation of Anhui University of Chinese Medicine (Grant No. 2020sjzd03), and University Natural Science Research Project of Anhui Province (No. KJ2019A0431).

## Conflict of Interest

The authors declare that the research was conducted in the absence of any commercial or financial relationships that could be construed as a potential conflict of interest.
