# Supplementary figures and images for: Thymoquinone Prevents Dopaminergic Neurodegeneration by Attenuating Oxidative Stress Via the Nrf2/ARE Pathway
Source: Front Pharmacol. 2021 Jan 14;11:615598. doi: 10.3389/fphar.2020.615598 (PMC7840486; doi:10.3389/fphar.2020.615598)

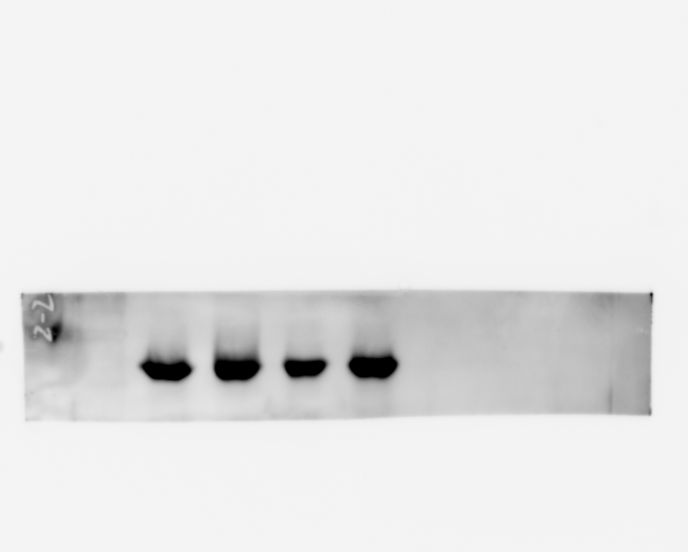

Supplement: Supplementary file 1 [file datasheet1.zip › supplementary material2/figure4C/TH-ms-1.tif]

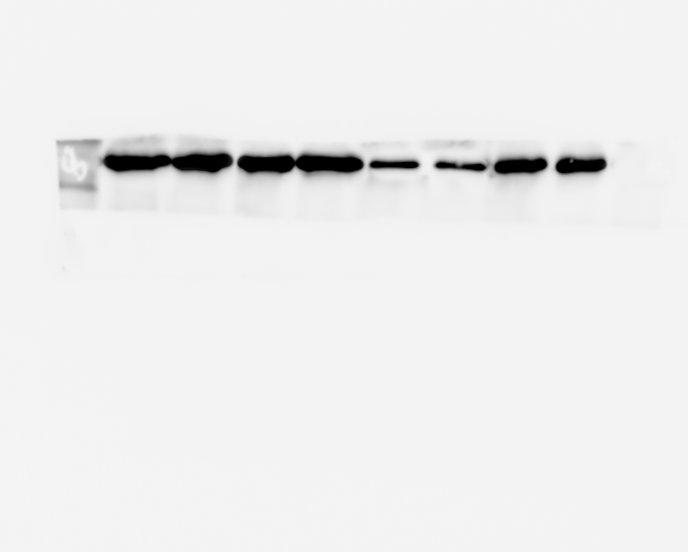

Supplement: Supplementary file 1 [file datasheet1.zip › supplementary material2/figure4C/TH-ms-2.tif]

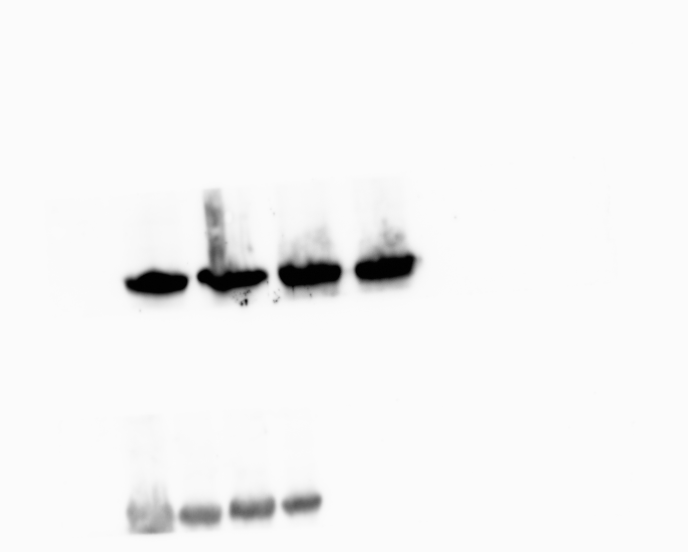

Supplement: Supplementary file 1 [file datasheet1.zip › supplementary material2/figure4C/actin-ms-1.tif]

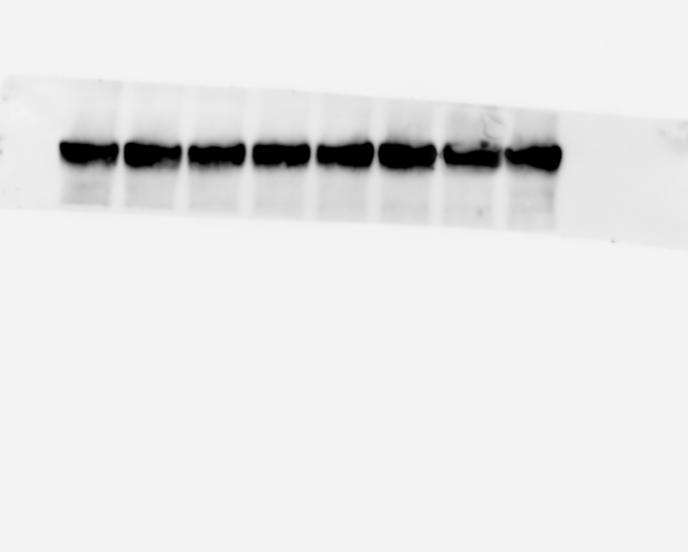

Supplement: Supplementary file 1 [file datasheet1.zip › supplementary material2/figure4C/actin-ms-2.tif]

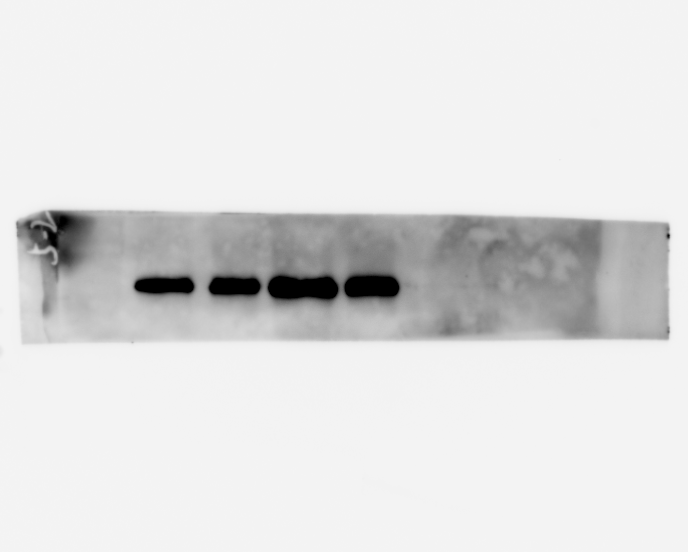

Supplement: Supplementary file 1 [file datasheet1.zip › supplementary material2/figure4C/syn-ms-1.tif]

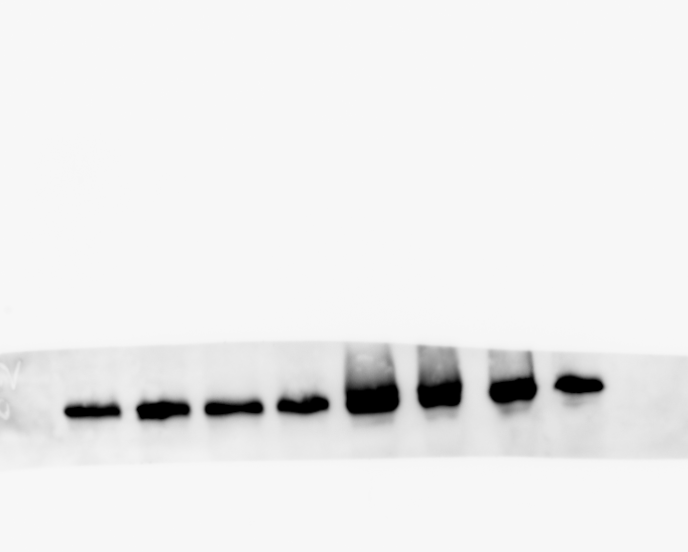

Supplement: Supplementary file 1 [file datasheet1.zip › supplementary material2/figure4C/syn-ms-2.tif]

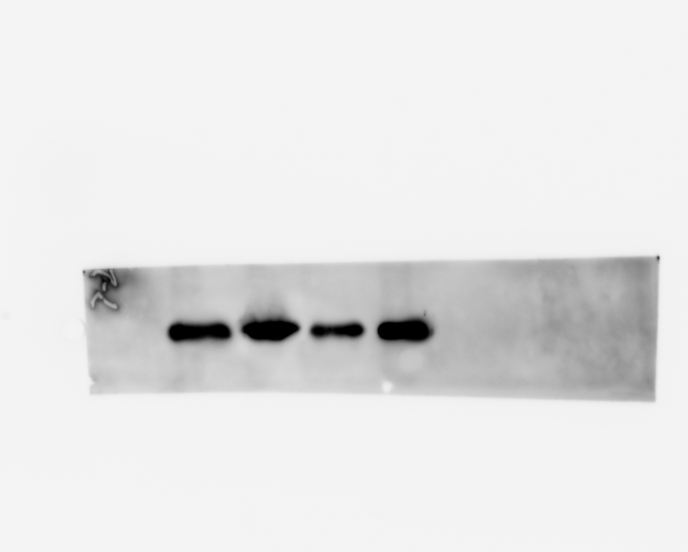

Supplement: Supplementary file 1 [file datasheet1.zip › supplementary material2/figure5E/GST-ms-1.tif]

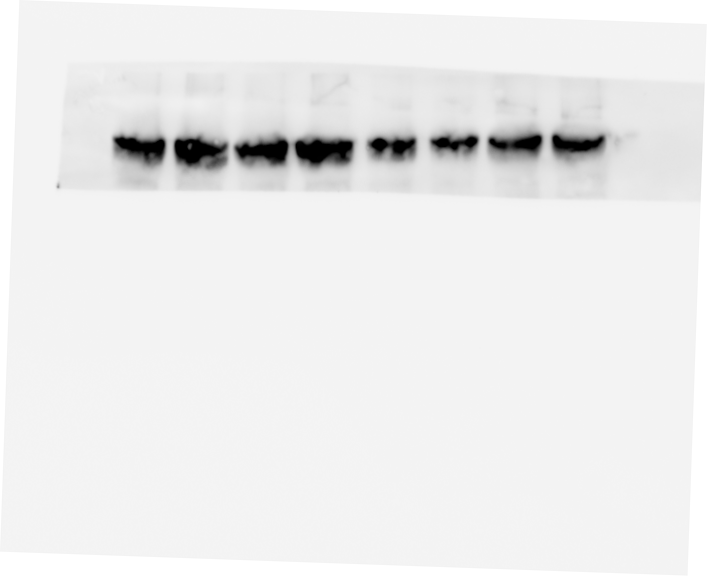

Supplement: Supplementary file 1 [file datasheet1.zip › supplementary material2/figure5E/GST-ms-2.tif]

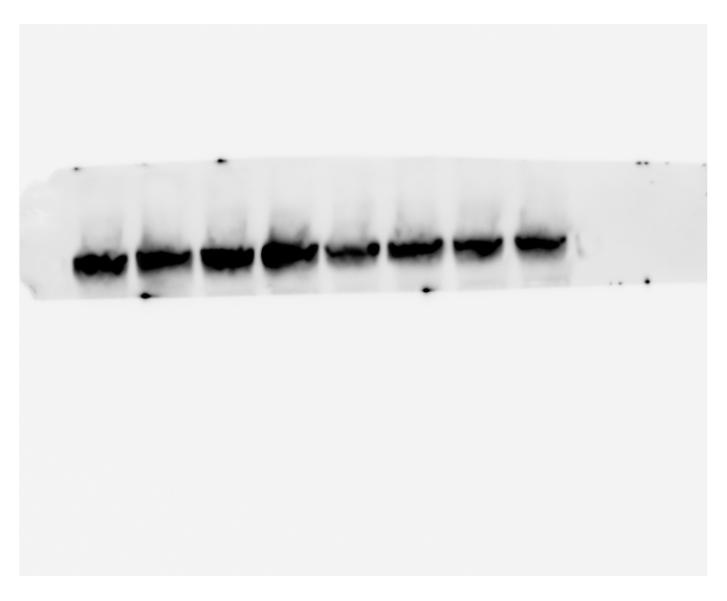

Supplement: Supplementary file 1 [file datasheet1.zip › supplementary material2/figure5E/GST-ms-3.tif]

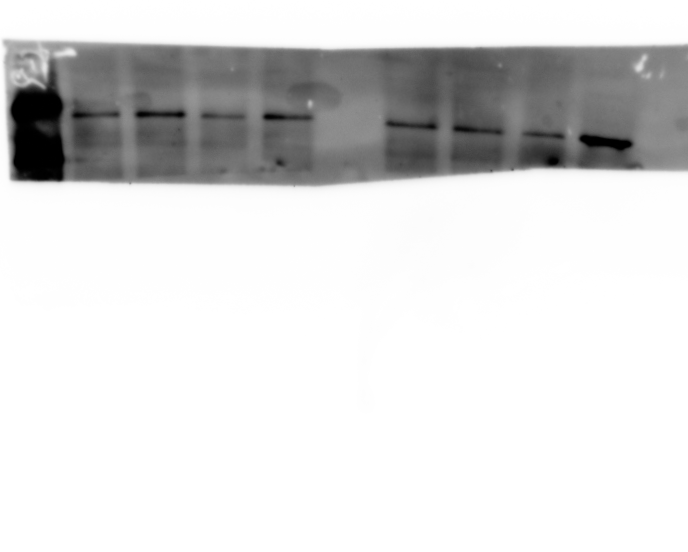

Supplement: Supplementary file 1 [file datasheet1.zip › supplementary material2/figure5E/HO1-ms-1.tif]

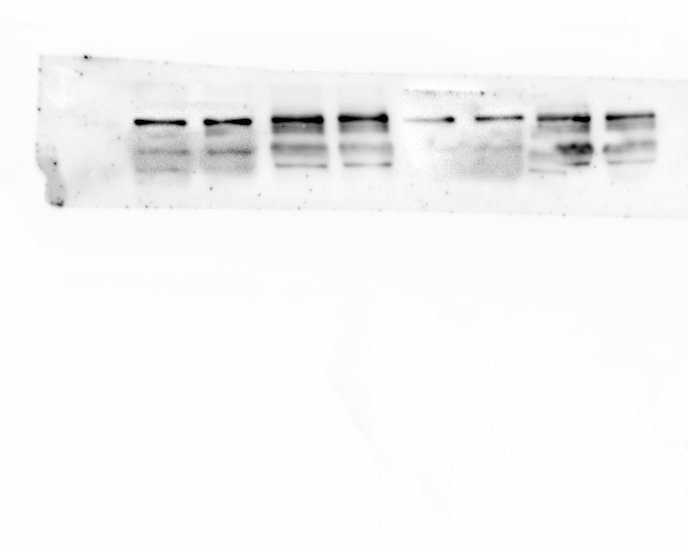

Supplement: Supplementary file 1 [file datasheet1.zip › supplementary material2/figure5E/HO1-ms-2.tif]

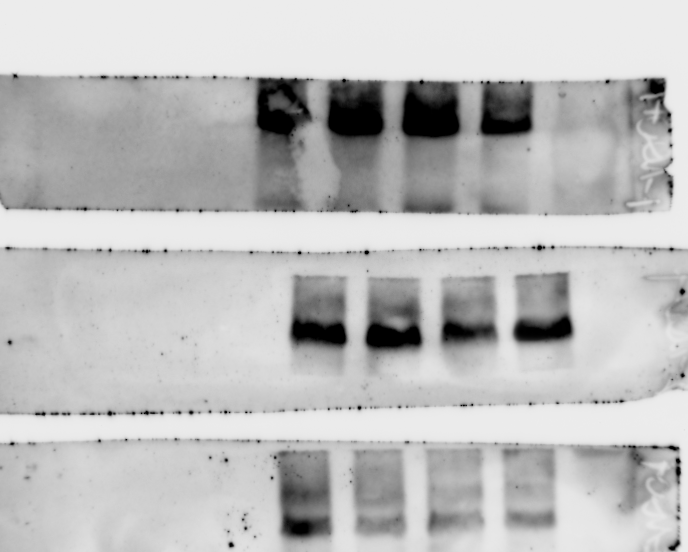

Supplement: Supplementary file 1 [file datasheet1.zip › supplementary material2/figure5E/NQO1-ms-1.tif]

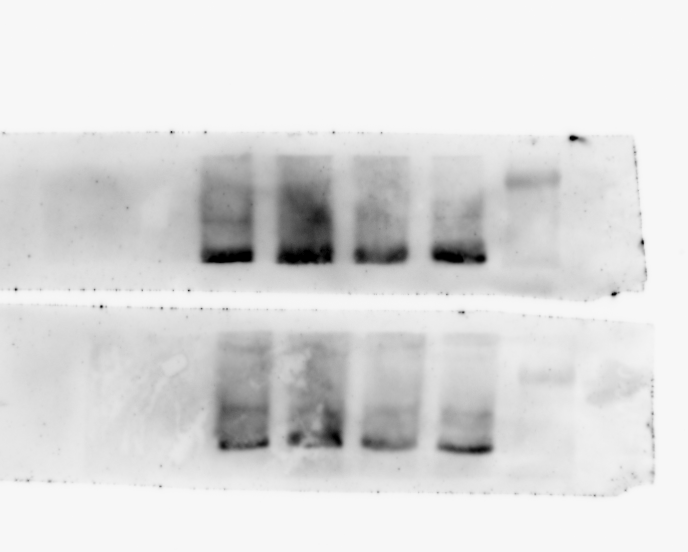

Supplement: Supplementary file 1 [file datasheet1.zip › supplementary material2/figure5E/NQO1-ms-2.tif]

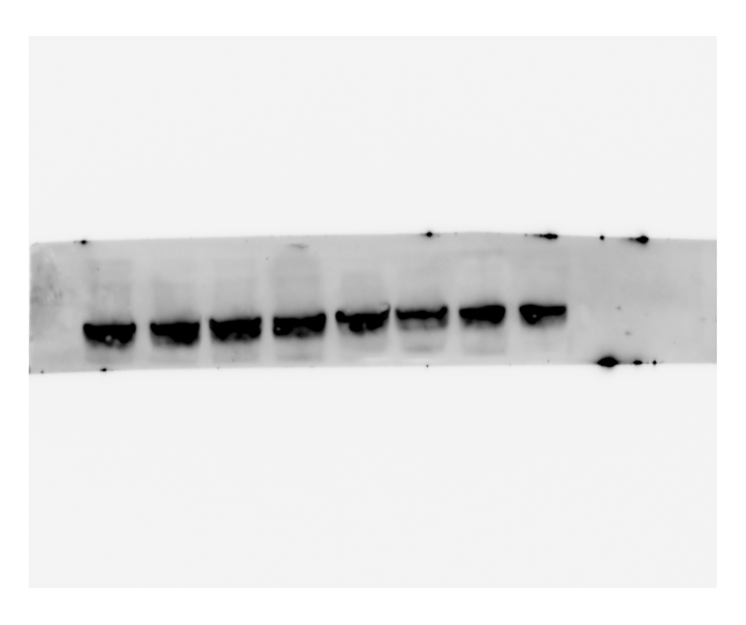

Supplement: Supplementary file 1 [file datasheet1.zip › supplementary material2/figure5E/NQO1-ms-3.tif]

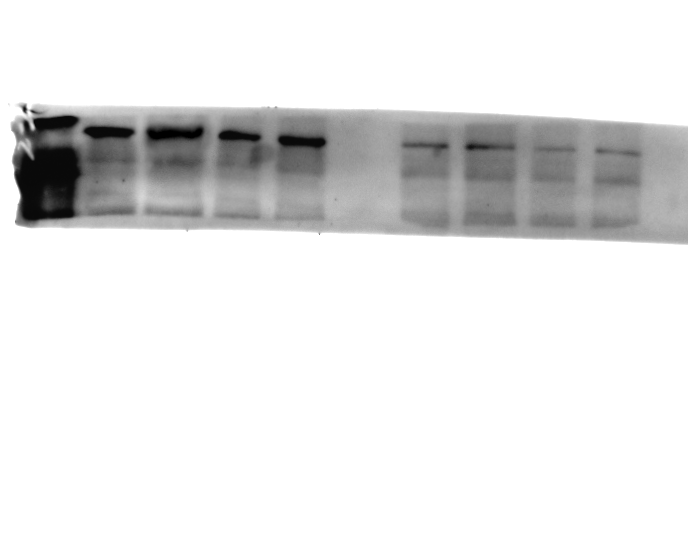

Supplement: Supplementary file 1 [file datasheet1.zip › supplementary material2/figure5E/Nrf2-ms-1.tif]

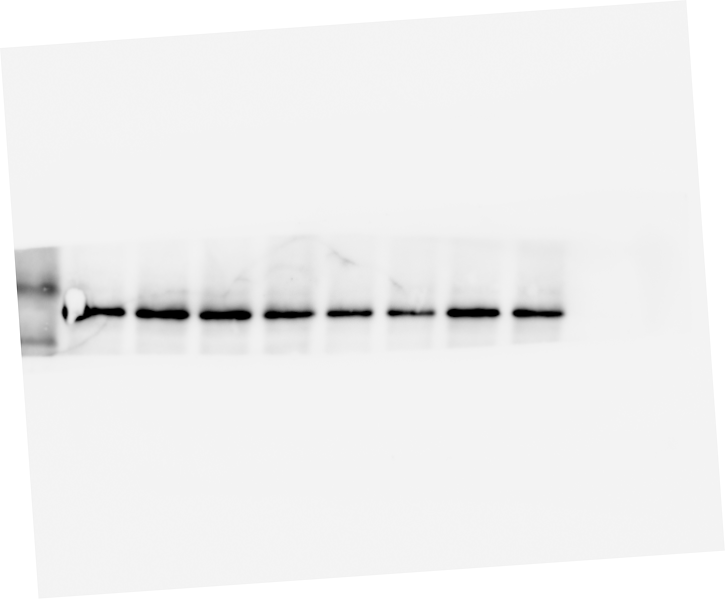

Supplement: Supplementary file 1 [file datasheet1.zip › supplementary material2/figure5E/Nrf2-ms-2.tif]

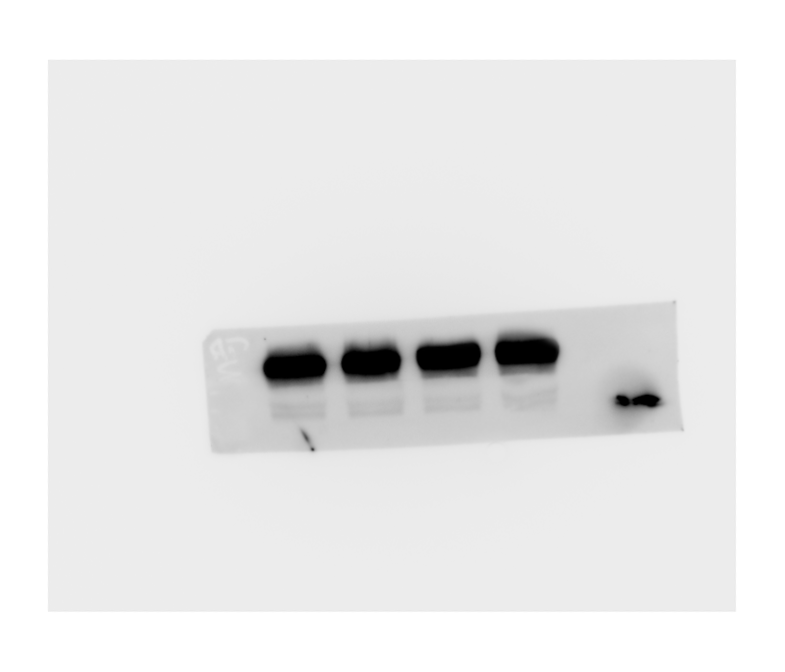

Supplement: Supplementary file 1 [file datasheet1.zip › supplementary material2/figure5E/actin-ms-3.tif]

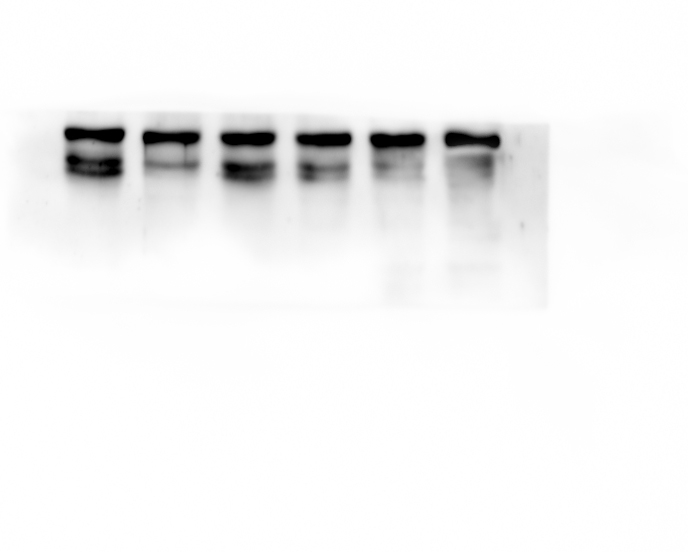

Supplement: Supplementary file 1 [file datasheet1.zip › supplementary material2/figure6B/siNrf2-actin-ms.tif]

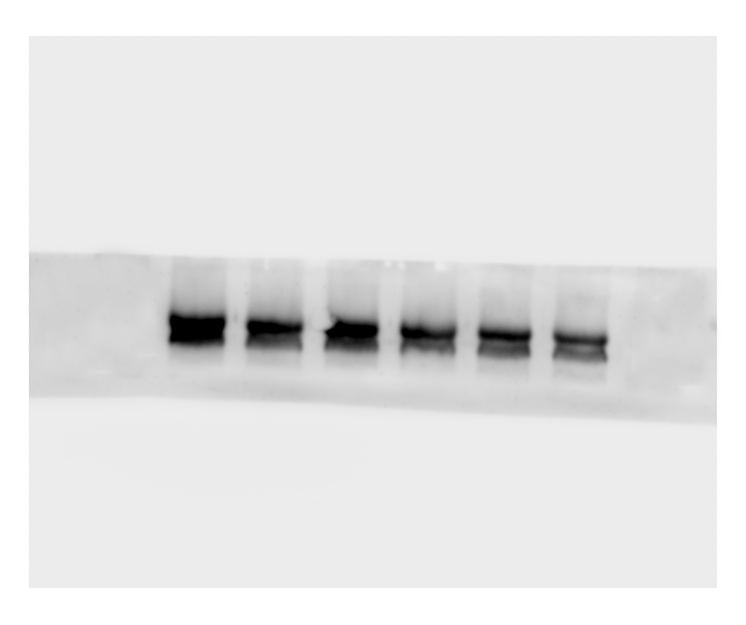

Supplement: Supplementary file 1 [file datasheet1.zip › supplementary material2/figure6B/siNrf2-ms.tif]

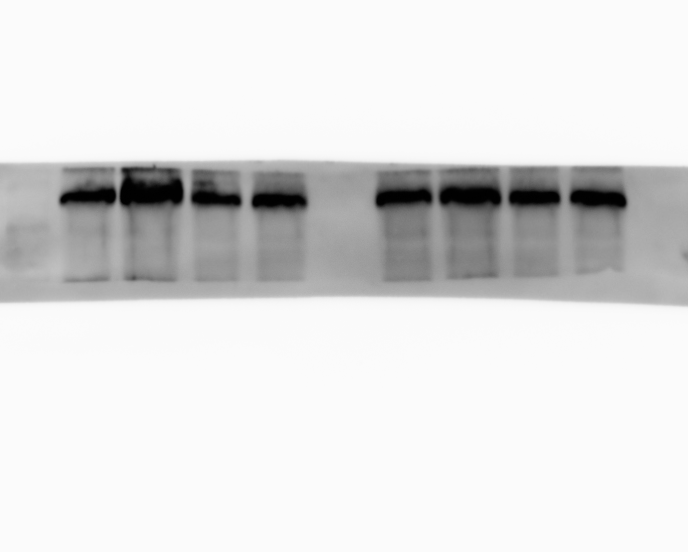

Supplement: Supplementary file 1 [file datasheet1.zip › supplementary material2/figure6C/si-GST-ms-1.tif]

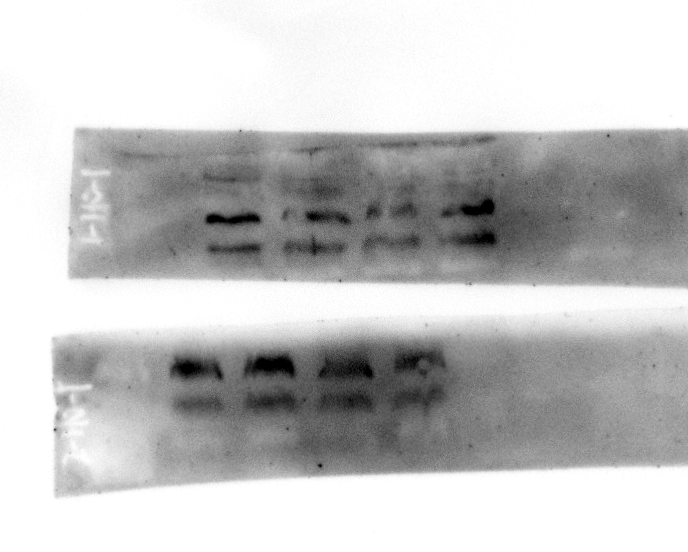

Supplement: Supplementary file 1 [file datasheet1.zip › supplementary material2/figure6C/si-HO1-ms-1.tif]

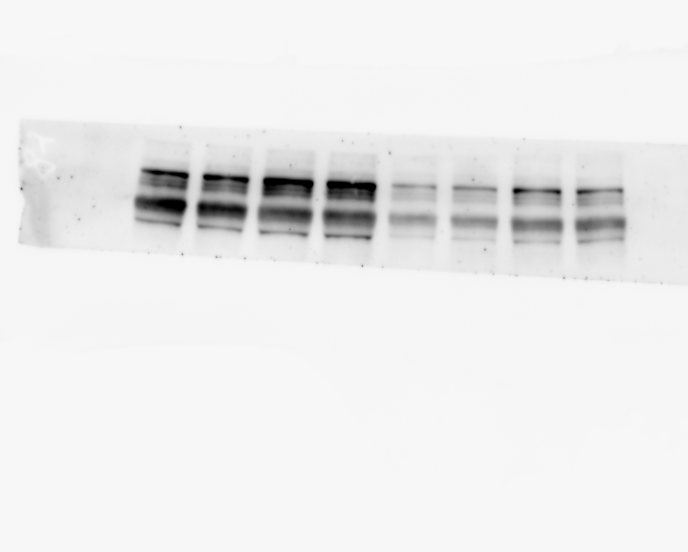

Supplement: Supplementary file 1 [file datasheet1.zip › supplementary material2/figure6C/si-HO1-ms-2.tif]

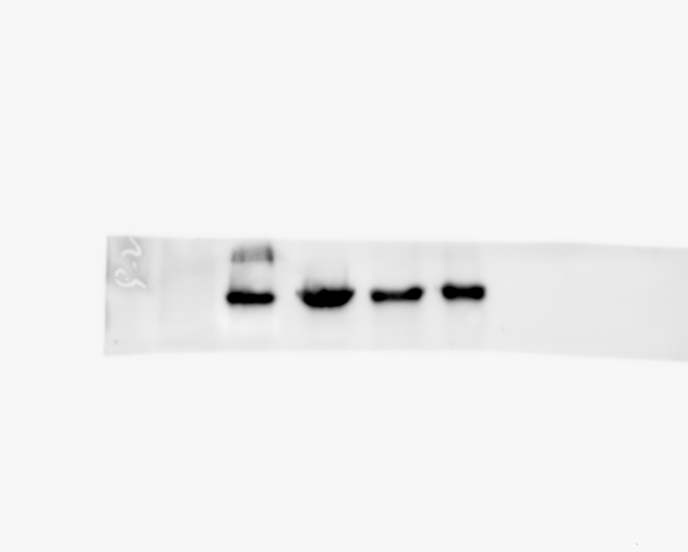

Supplement: Supplementary file 1 [file datasheet1.zip › supplementary material2/figure6C/si-NQO1-ms-1.tif]

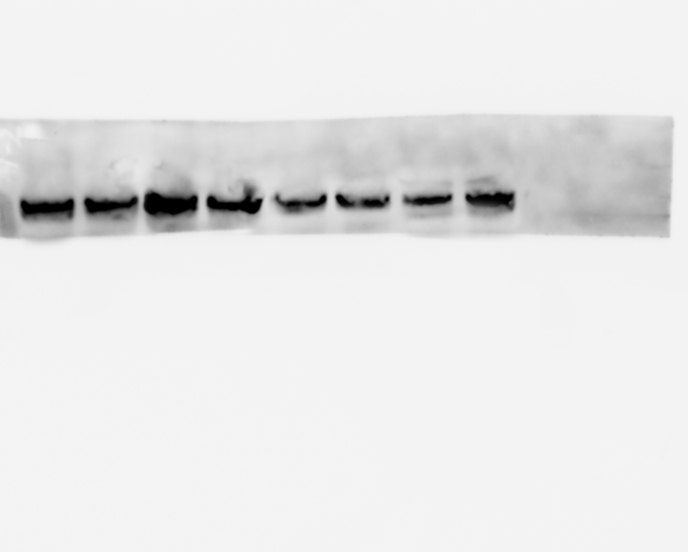

Supplement: Supplementary file 1 [file datasheet1.zip › supplementary material2/figure6C/si-NQO1-ms-2.tif]

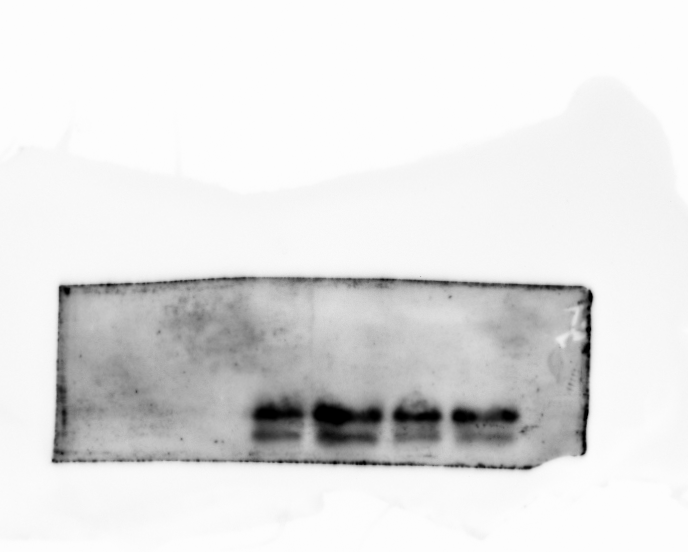

Supplement: Supplementary file 1 [file datasheet1.zip › supplementary material2/figure6C/si-Nrf2-ms-1.tif]

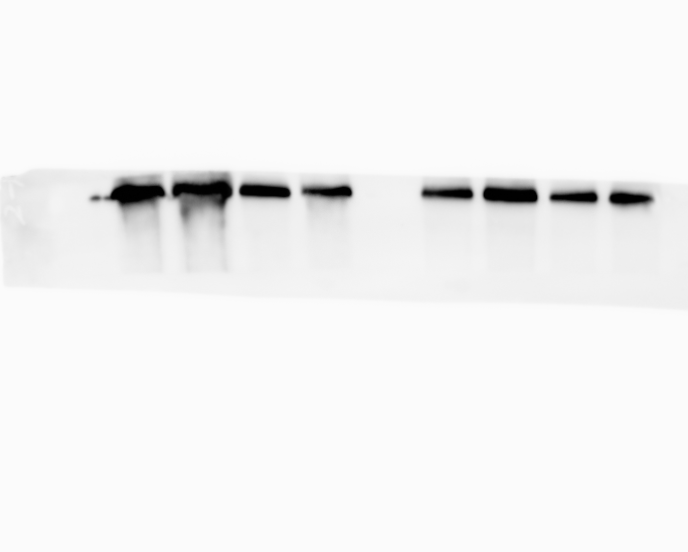

Supplement: Supplementary file 1 [file datasheet1.zip › supplementary material2/figure6C/si-Nrf2-ms-2.tif]

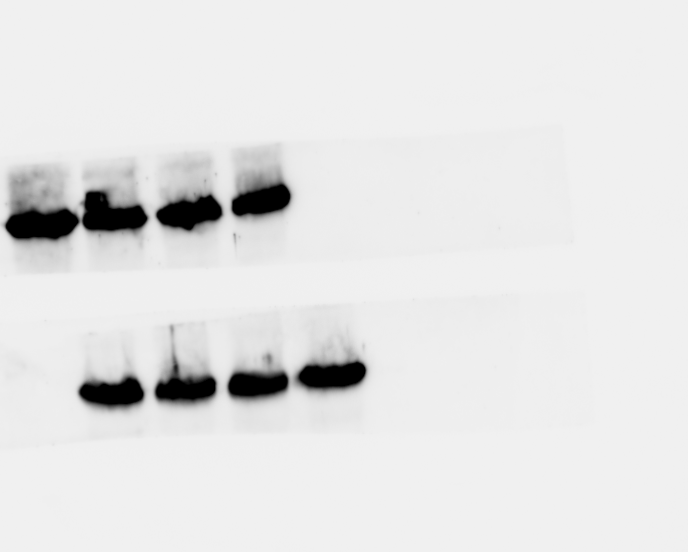

Supplement: Supplementary file 1 [file datasheet1.zip › supplementary material2/figure6C/si-actin-ms-1.tif]

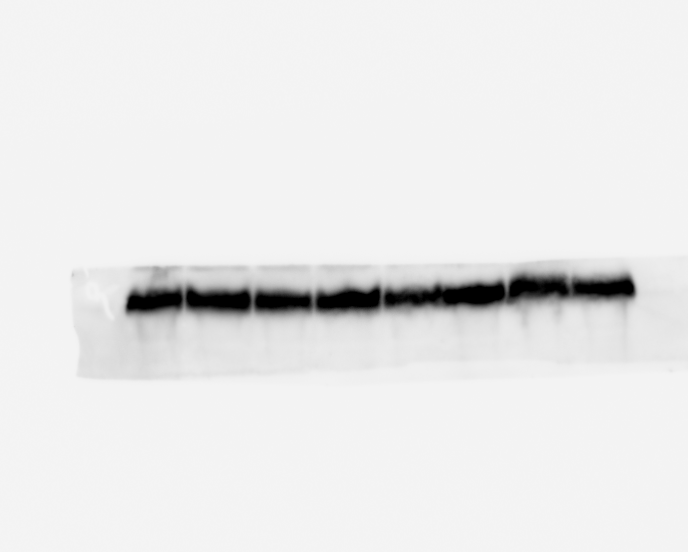

Supplement: Supplementary file 1 [file datasheet1.zip › supplementary material2/figure6C/si-actin-ms-2.tif]

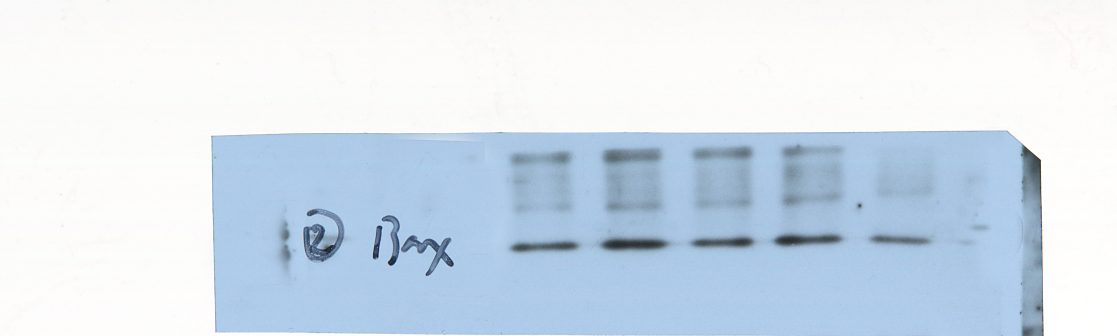

Supplement: Supplementary file 2 [file datasheet2.zip › supplementary material1/Figure1E/BAX-cell-1.tif]

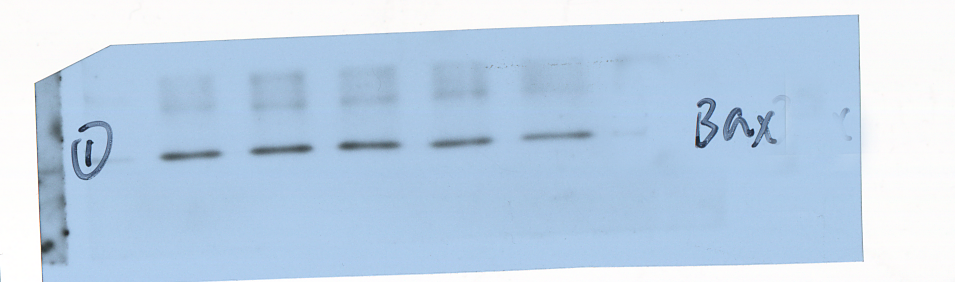

Supplement: Supplementary file 2 [file datasheet2.zip › supplementary material1/Figure1E/BAX-cell-2.tif]

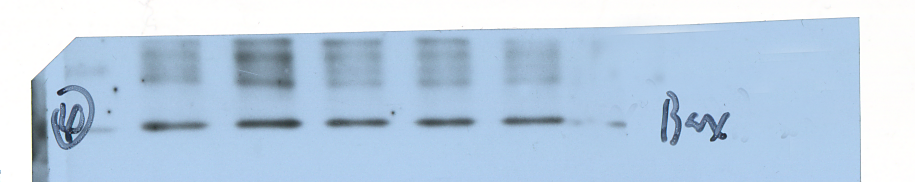

Supplement: Supplementary file 2 [file datasheet2.zip › supplementary material1/Figure1E/BAX-cell-3.tif]

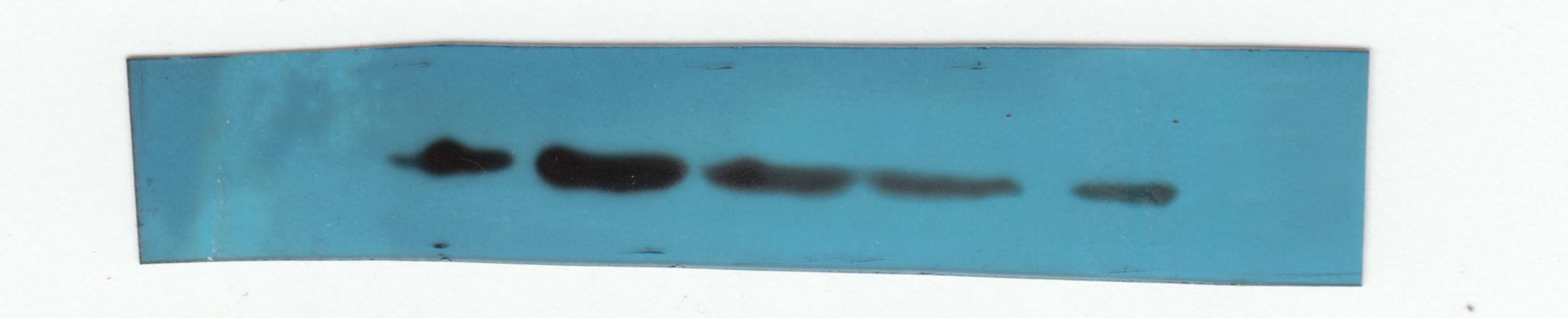

Supplement: Supplementary file 2 [file datasheet2.zip › supplementary material1/Figure1E/Casp3-cell-1.tif]

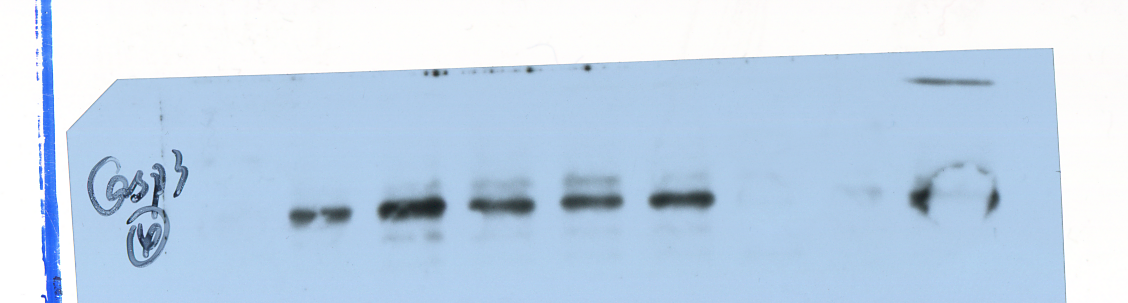

Supplement: Supplementary file 2 [file datasheet2.zip › supplementary material1/Figure1E/Casp3-cell-2.tif]

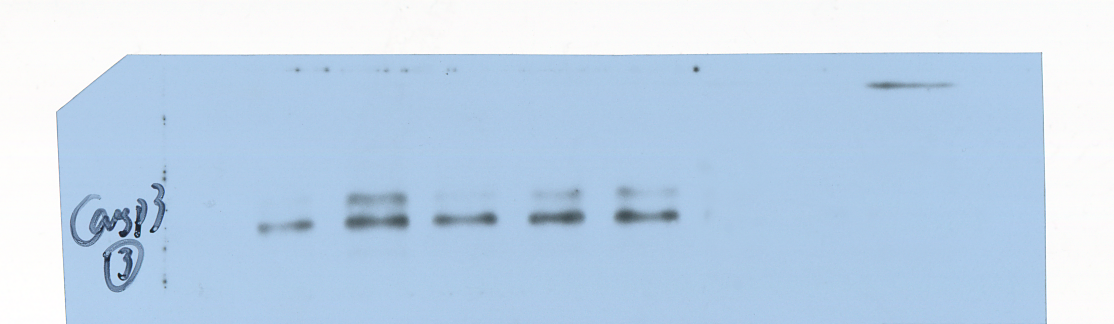

Supplement: Supplementary file 2 [file datasheet2.zip › supplementary material1/Figure1E/Casp3-cell-3.tif]

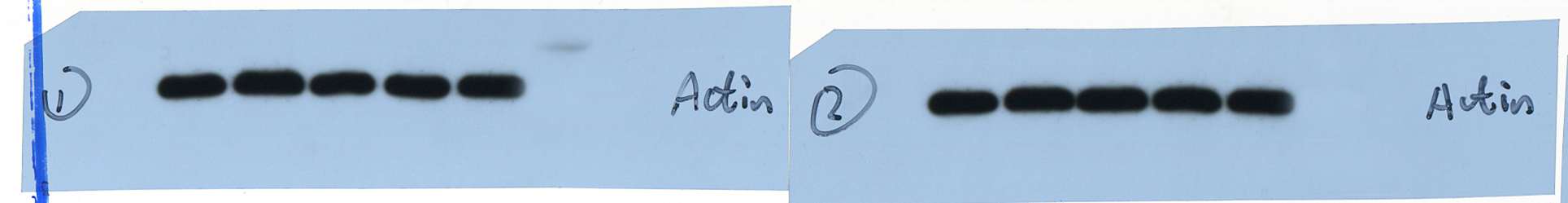

Supplement: Supplementary file 2 [file datasheet2.zip › supplementary material1/Figure1E/actin-cell-12.tif]

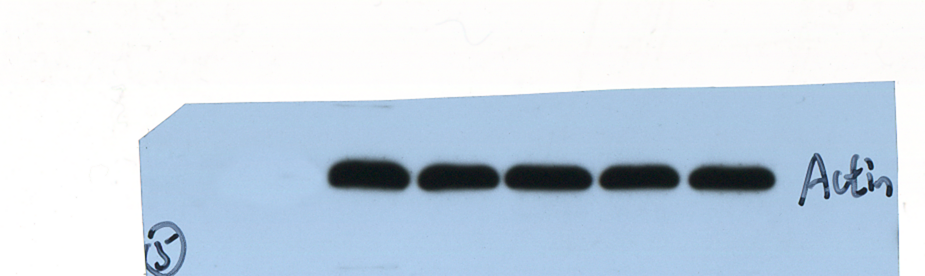

Supplement: Supplementary file 2 [file datasheet2.zip › supplementary material1/Figure1E/actin-cell-3.tif]

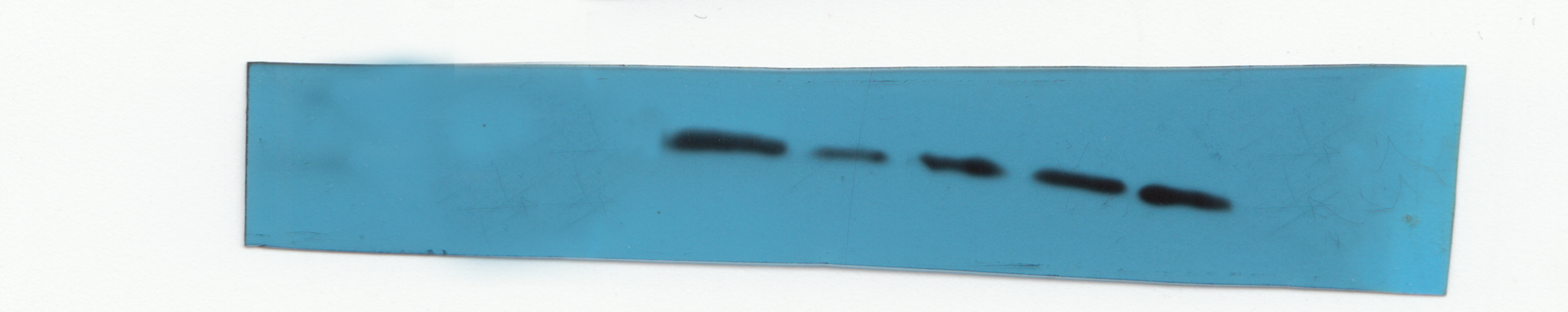

Supplement: Supplementary file 2 [file datasheet2.zip › supplementary material1/Figure1E/bcl2-cell-1.tif]

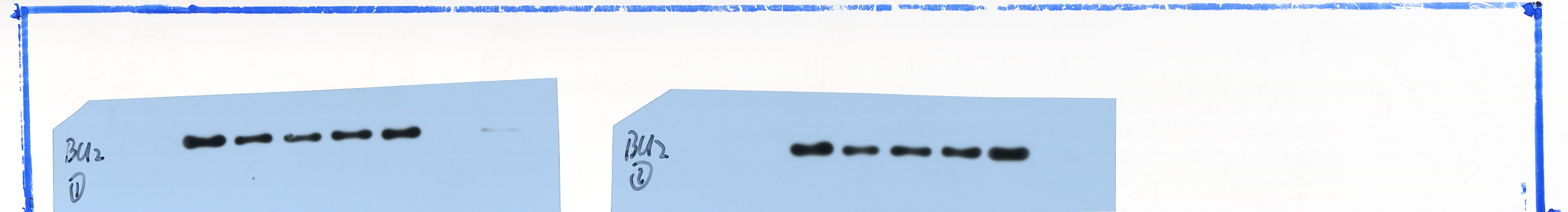

Supplement: Supplementary file 2 [file datasheet2.zip › supplementary material1/Figure1E/bcl2-cell-23.tif]

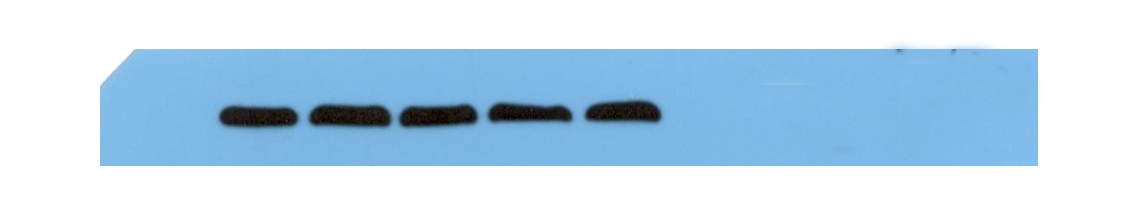

Supplement: Supplementary file 2 [file datasheet2.zip › supplementary material1/figure2E/LaminB1-1.tif]

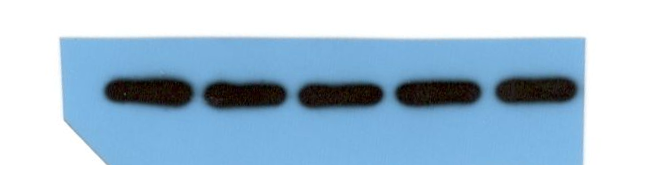

Supplement: Supplementary file 2 [file datasheet2.zip › supplementary material1/figure2E/LaminB1-2.tif]

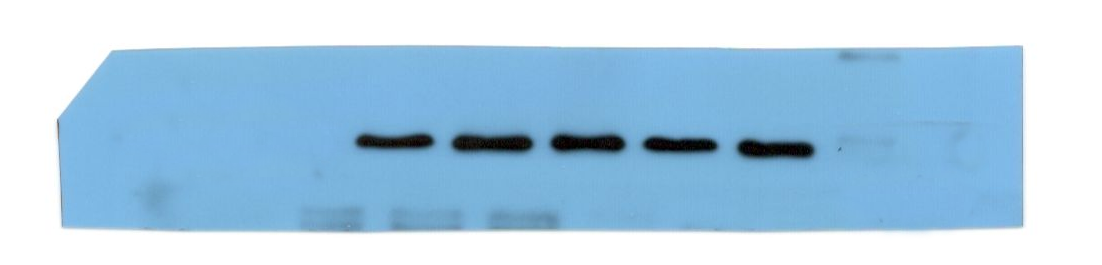

Supplement: Supplementary file 2 [file datasheet2.zip › supplementary material1/figure2E/LaminB1-3.tif]

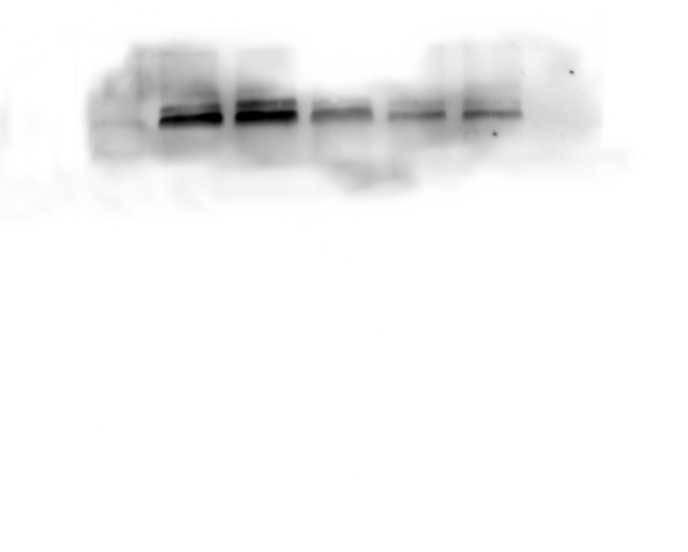

Supplement: Supplementary file 2 [file datasheet2.zip › supplementary material1/figure2E/cytoplasmic Nrf2-1.tif]

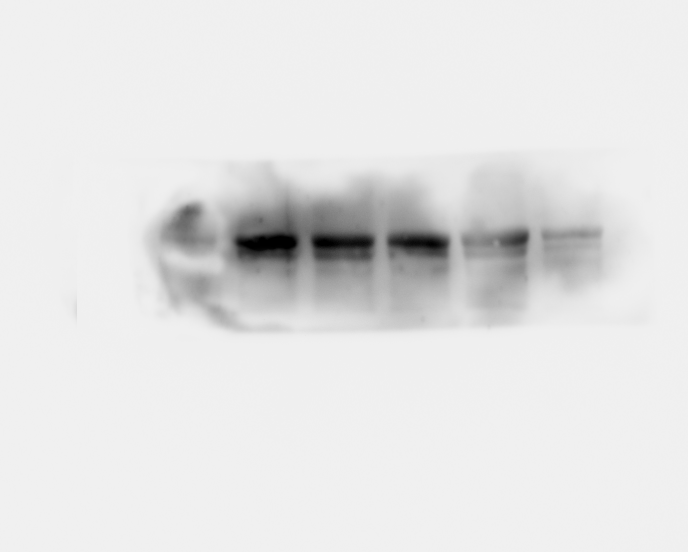

Supplement: Supplementary file 2 [file datasheet2.zip › supplementary material1/figure2E/cytoplasmic Nrf2-2.tif]

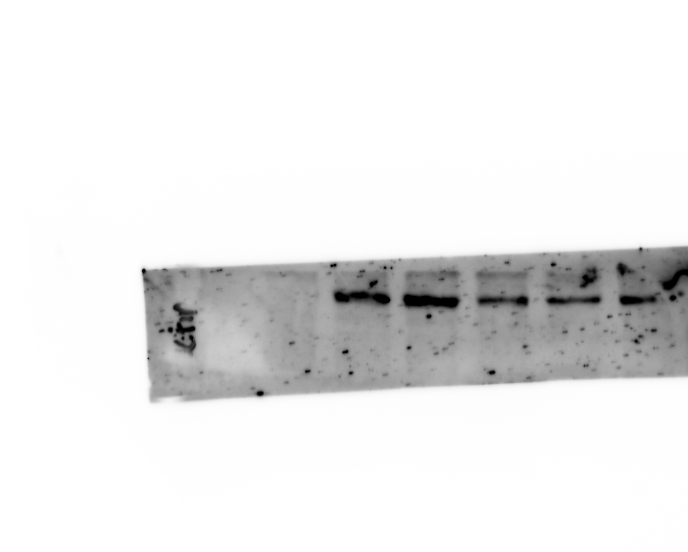

Supplement: Supplementary file 2 [file datasheet2.zip › supplementary material1/figure2E/cytoplasmic Nrf2-3.tif]

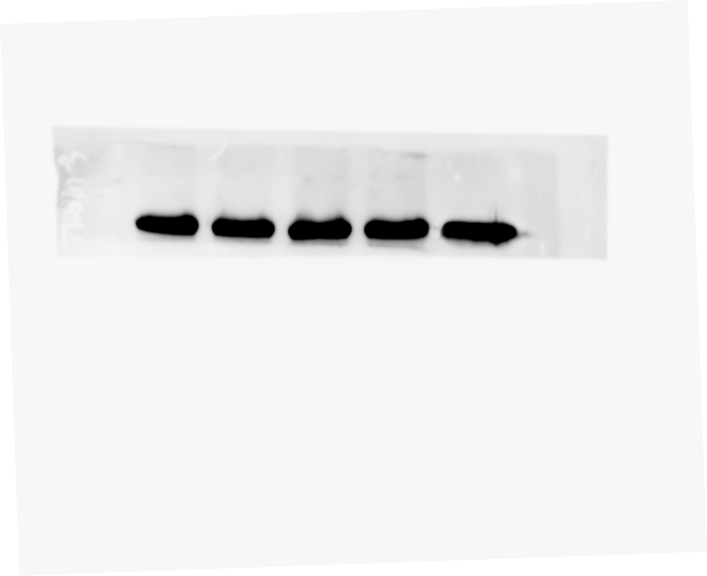

Supplement: Supplementary file 2 [file datasheet2.zip › supplementary material1/figure2E/cytoplasmic-actin-1.tif]

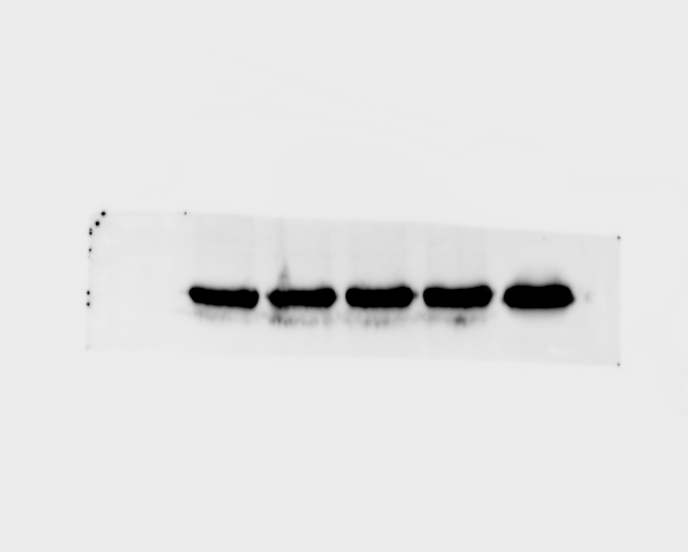

Supplement: Supplementary file 2 [file datasheet2.zip › supplementary material1/figure2E/cytoplasmic-actin-2.tif]

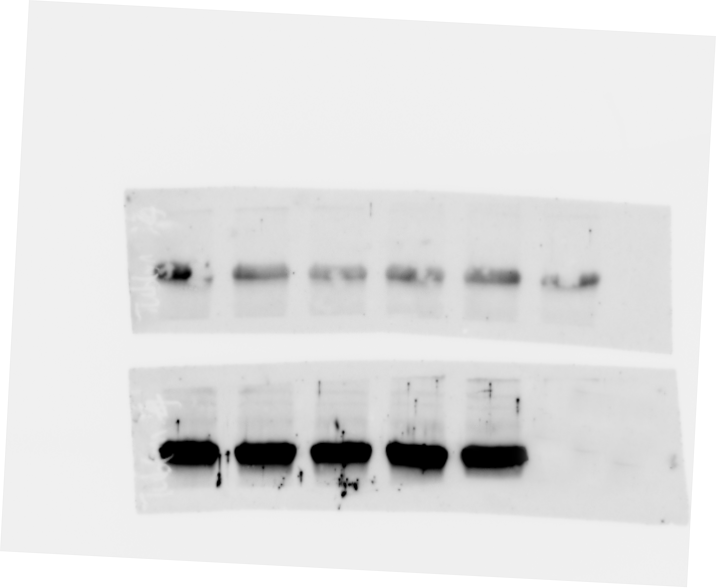

Supplement: Supplementary file 2 [file datasheet2.zip › supplementary material1/figure2E/cytoplasmic-actin-3.tif]

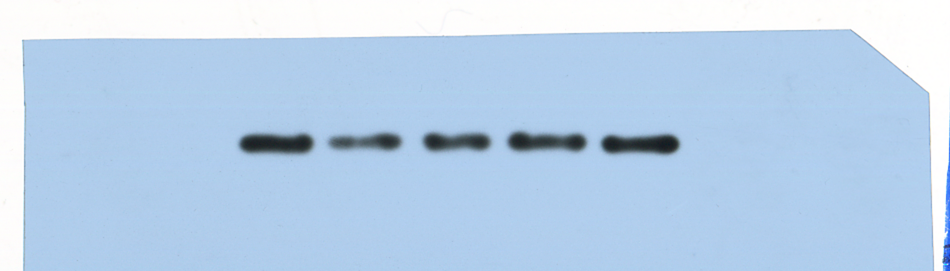

Supplement: Supplementary file 2 [file datasheet2.zip › supplementary material1/figure2E/cytosolic-Nrf2-1.tif]

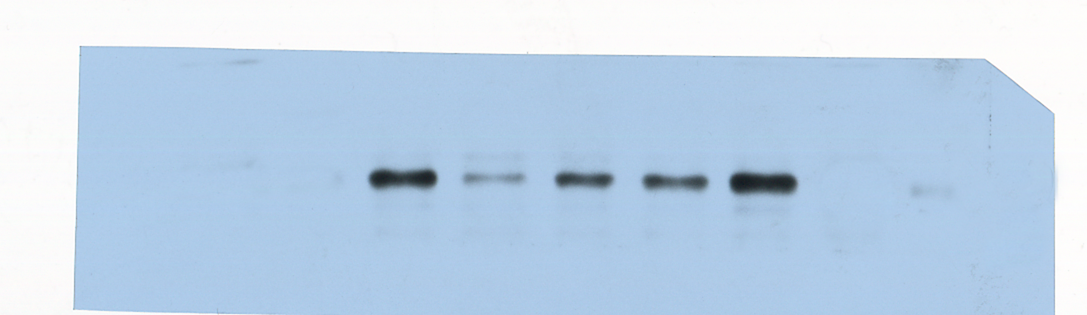

Supplement: Supplementary file 2 [file datasheet2.zip › supplementary material1/figure2E/cytosolic-Nrf2-2.tif]

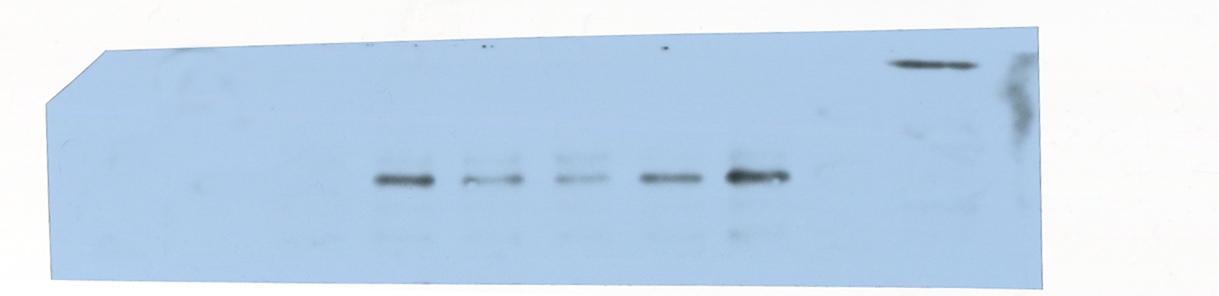

Supplement: Supplementary file 2 [file datasheet2.zip › supplementary material1/figure2E/cytosolic-Nrf2-3.tif]

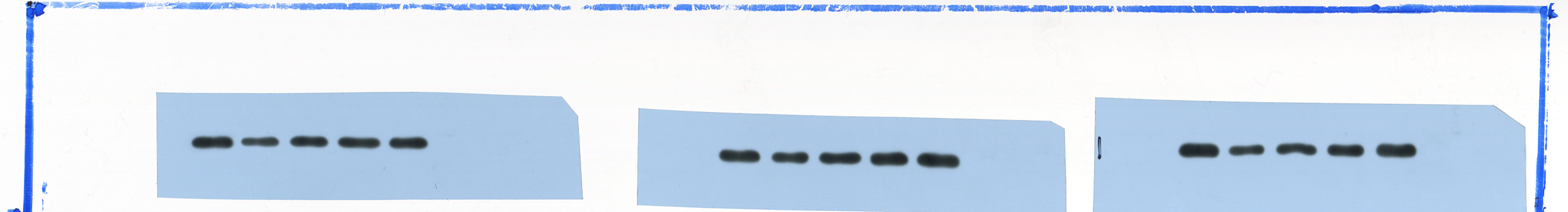

Supplement: Supplementary file 2 [file datasheet2.zip › supplementary material1/figure2G/GST-cell.tif]

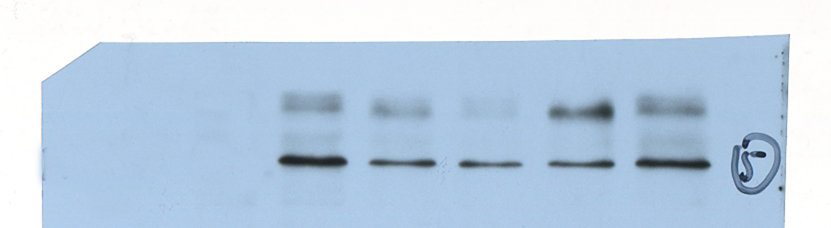

Supplement: Supplementary file 2 [file datasheet2.zip › supplementary material1/figure2G/HO1-cell-1.tif]

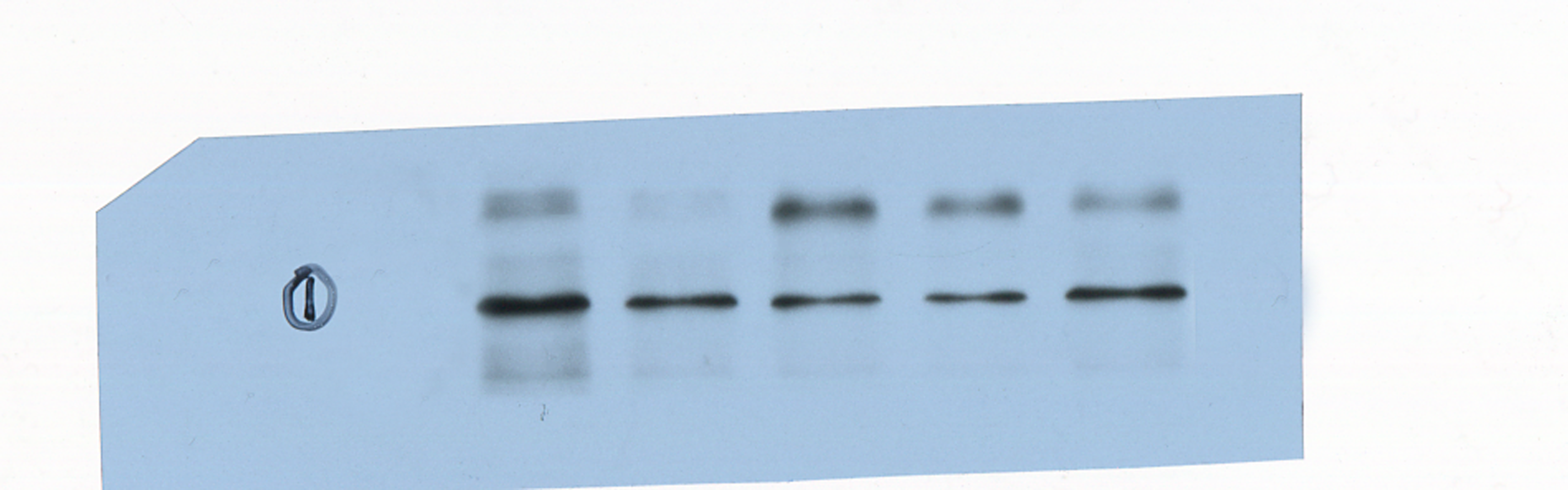

Supplement: Supplementary file 2 [file datasheet2.zip › supplementary material1/figure2G/HO1-cell-2.tif]

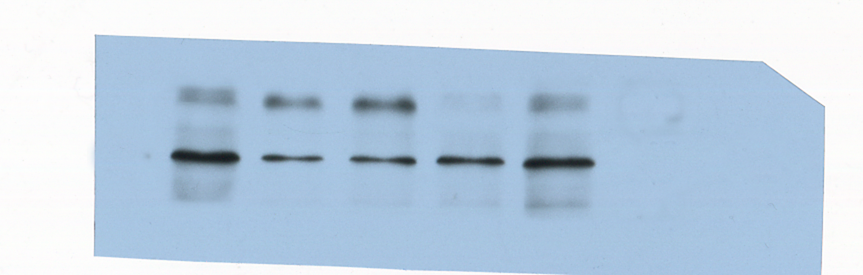

Supplement: Supplementary file 2 [file datasheet2.zip › supplementary material1/figure2G/HO1-cell-3.tif]

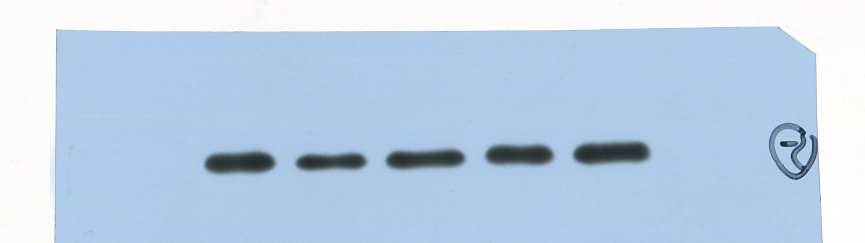

Supplement: Supplementary file 2 [file datasheet2.zip › supplementary material1/figure2G/NQO1-cell-1.tif]

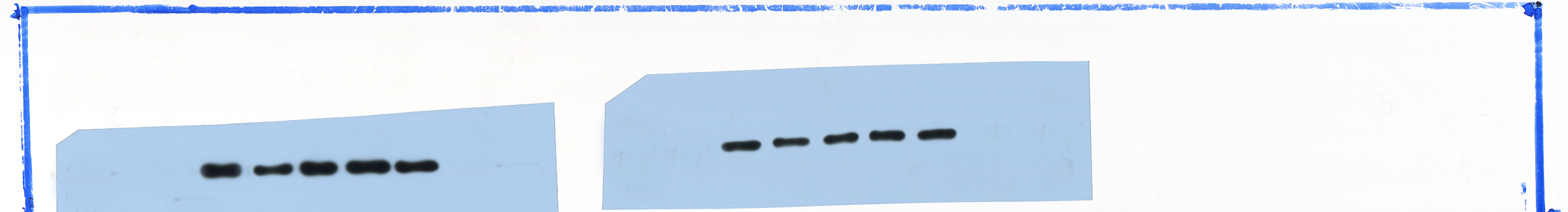

Supplement: Supplementary file 2 [file datasheet2.zip › supplementary material1/figure2G/NQO1-cell-23.tif]

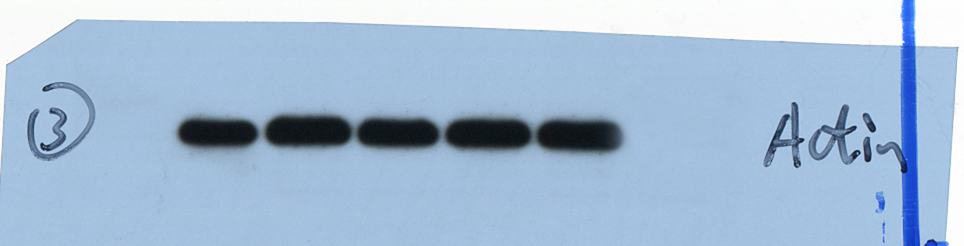

Supplement: Supplementary file 2 [file datasheet2.zip › supplementary material1/figure2G/actin-cell-oxi-1.tif]

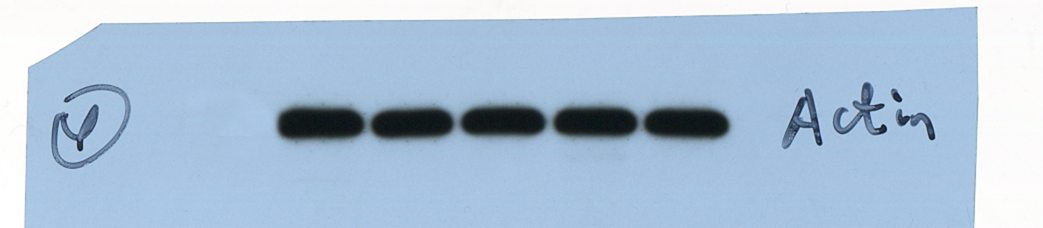

Supplement: Supplementary file 2 [file datasheet2.zip › supplementary material1/figure2G/actin-cell-oxi-2.tif]

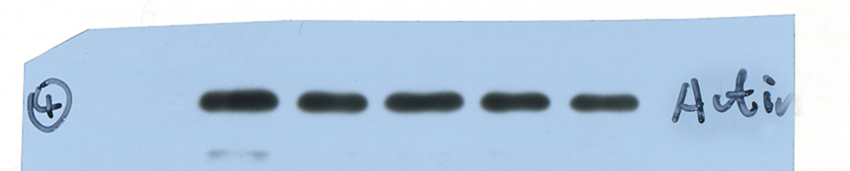

Supplement: Supplementary file 2 [file datasheet2.zip › supplementary material1/figure2G/actin-cell-oxi-3.tif]

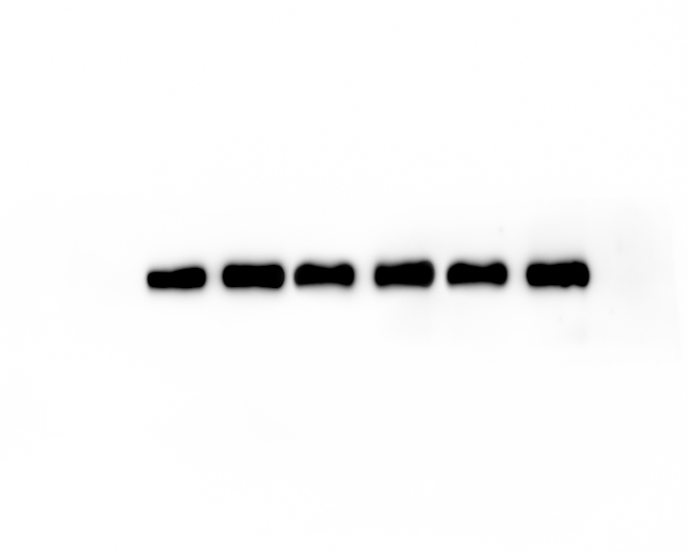

Supplement: Supplementary file 2 [file datasheet2.zip › supplementary material1/figure3A/siNrf2-actin-cell.tif]

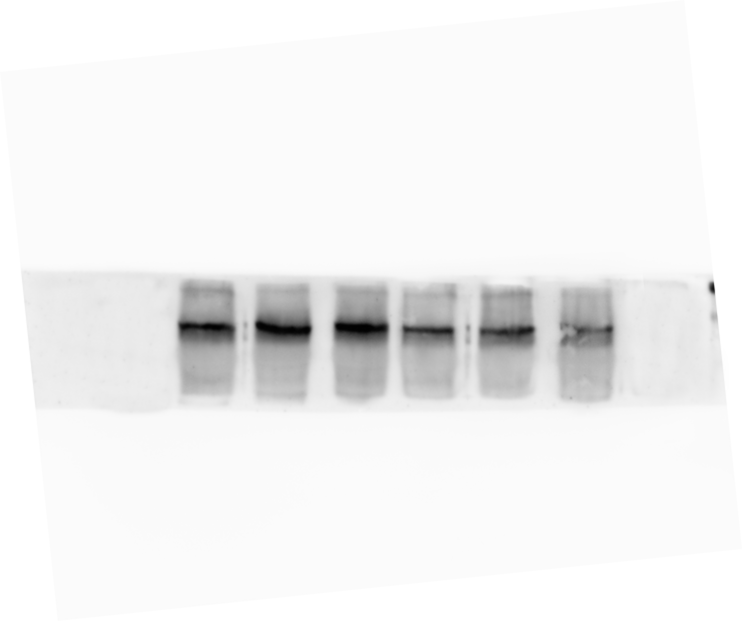

Supplement: Supplementary file 2 [file datasheet2.zip › supplementary material1/figure3A/siNrf2-cell.tif]

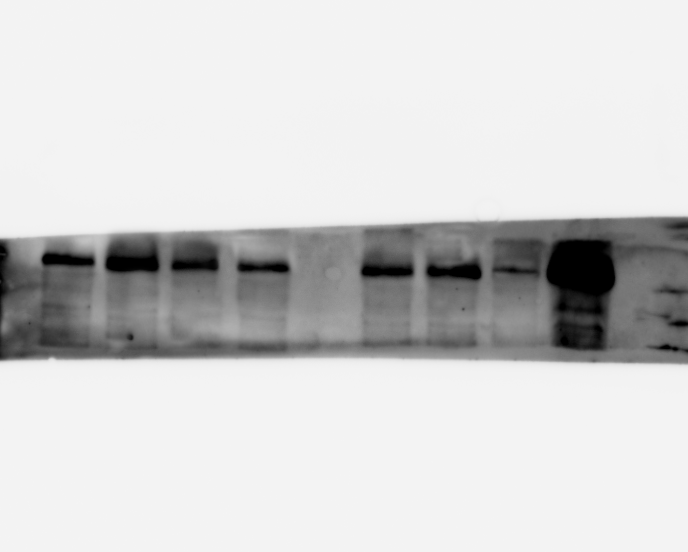

Supplement: Supplementary file 2 [file datasheet2.zip › supplementary material1/figure3C/si-Nrf2-cell-1.tif]

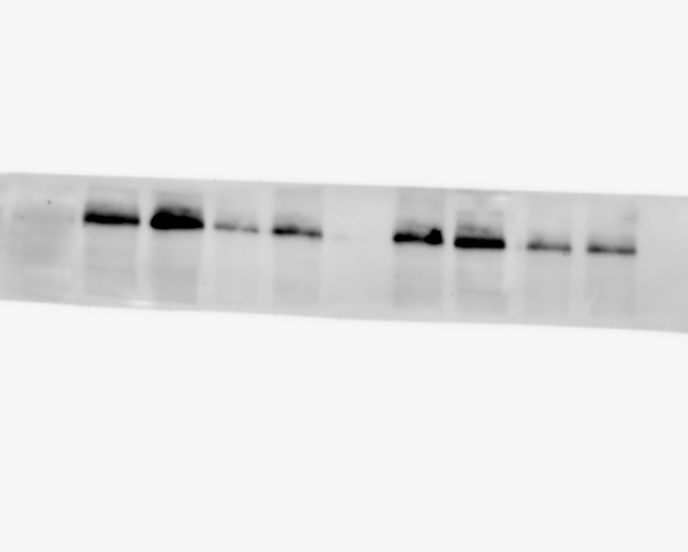

Supplement: Supplementary file 2 [file datasheet2.zip › supplementary material1/figure3C/si-Nrf2-cell-2.tif]

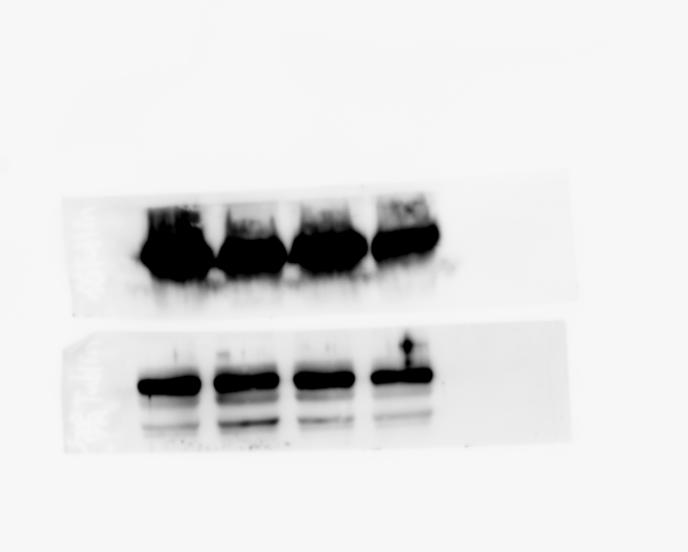

Supplement: Supplementary file 2 [file datasheet2.zip › supplementary material1/figure3C/si-laminB1-cell-1.tif]

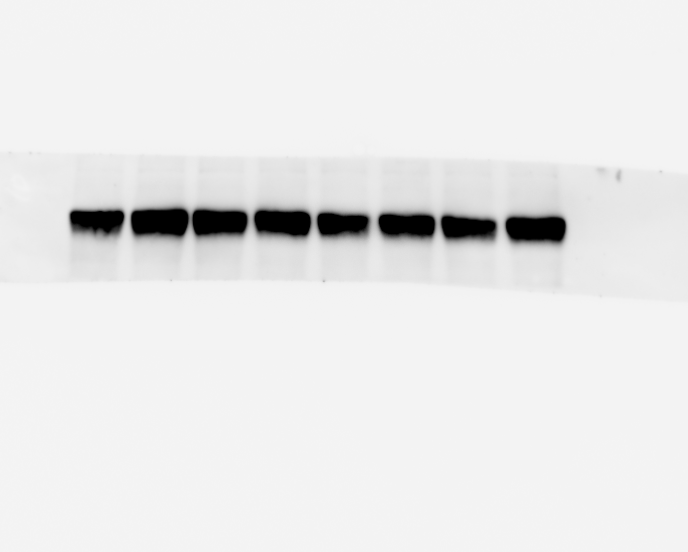

Supplement: Supplementary file 2 [file datasheet2.zip › supplementary material1/figure3C/si-laminB1-cell-2.tif]

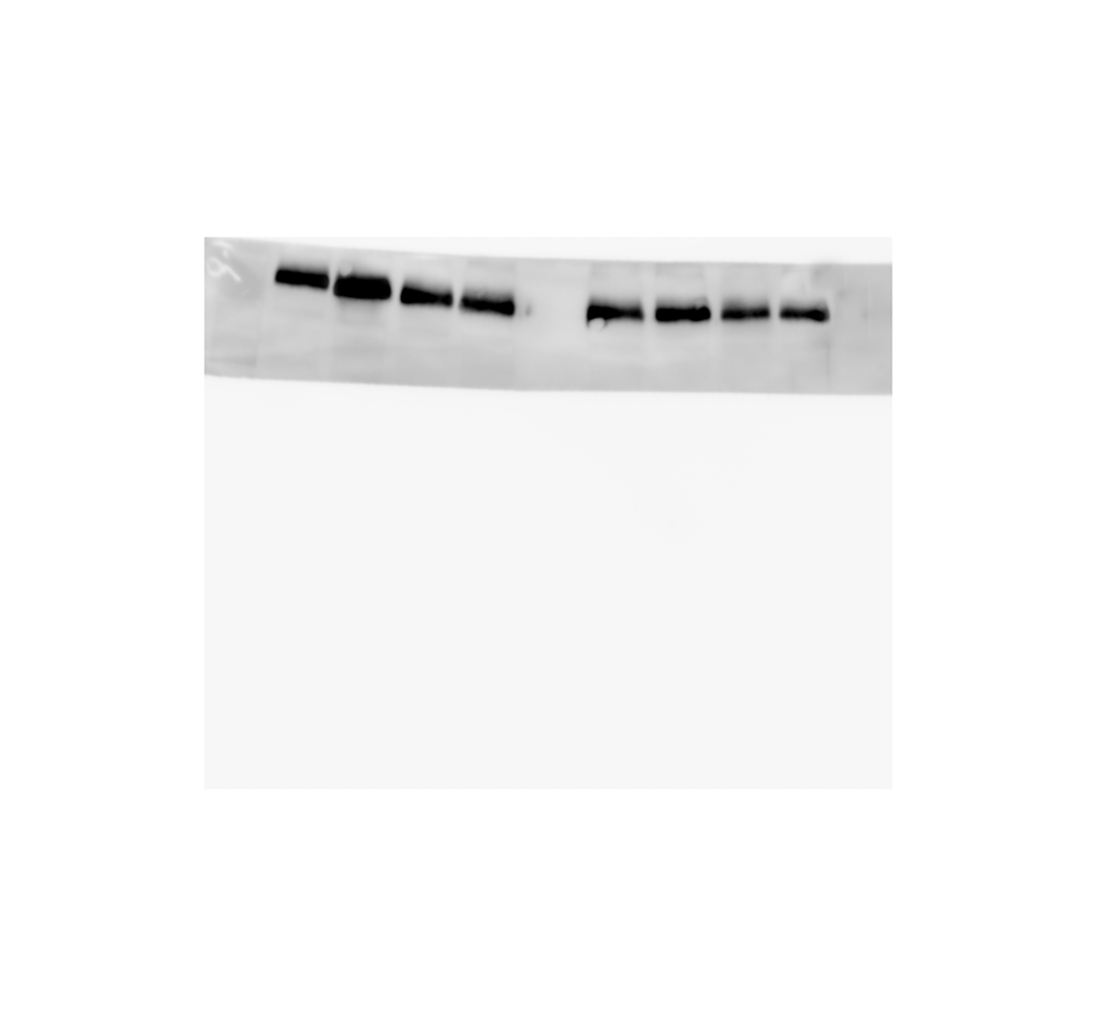

Supplement: Supplementary file 2 [file datasheet2.zip › supplementary material1/figure3D/si-GST-cell-1.tif]

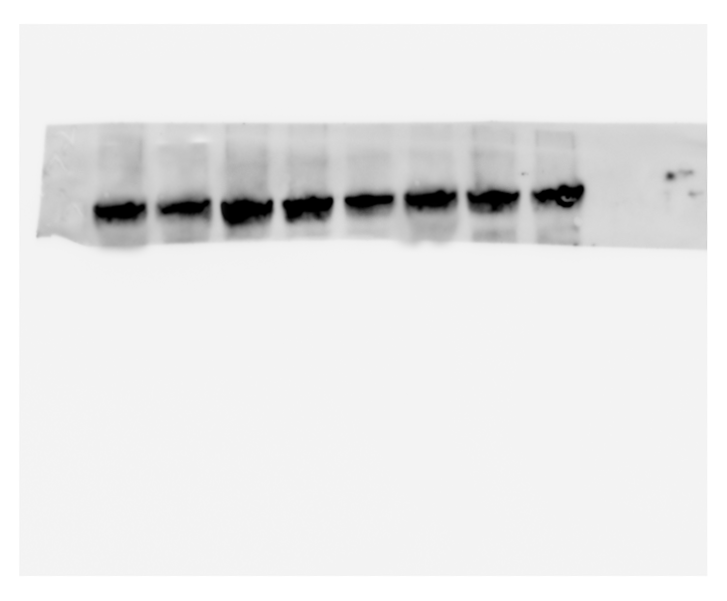

Supplement: Supplementary file 2 [file datasheet2.zip › supplementary material1/figure3D/si-GST-cell-2.tif]

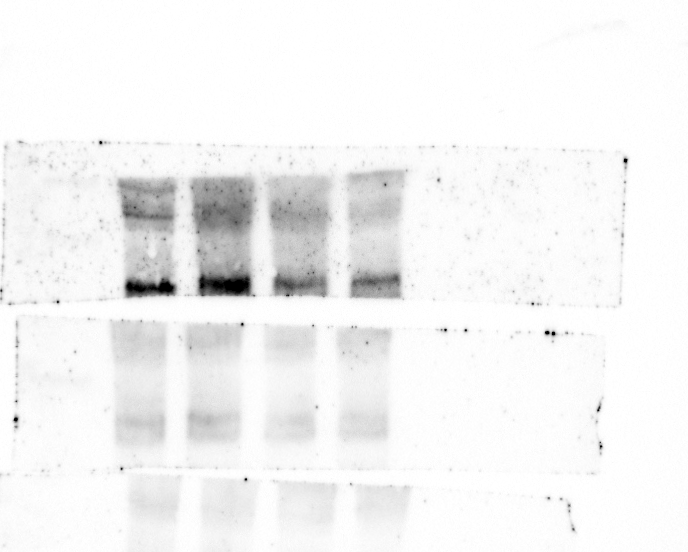

Supplement: Supplementary file 2 [file datasheet2.zip › supplementary material1/figure3D/si-HO1-cell-1.tif]

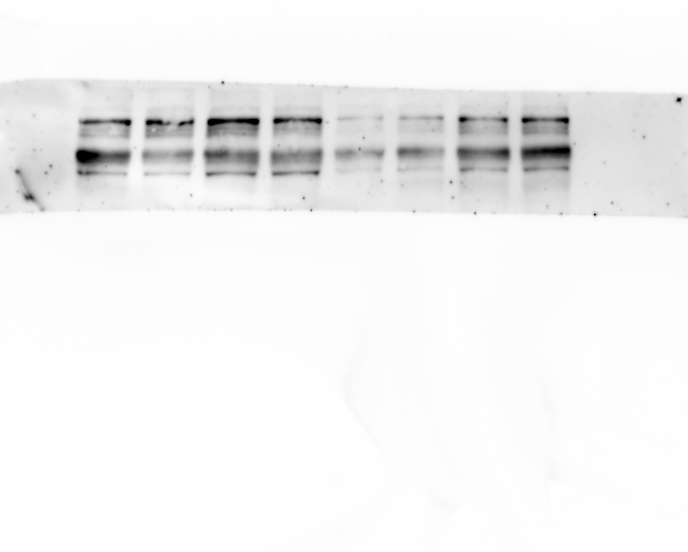

Supplement: Supplementary file 2 [file datasheet2.zip › supplementary material1/figure3D/si-HO1-cell-2.tif]

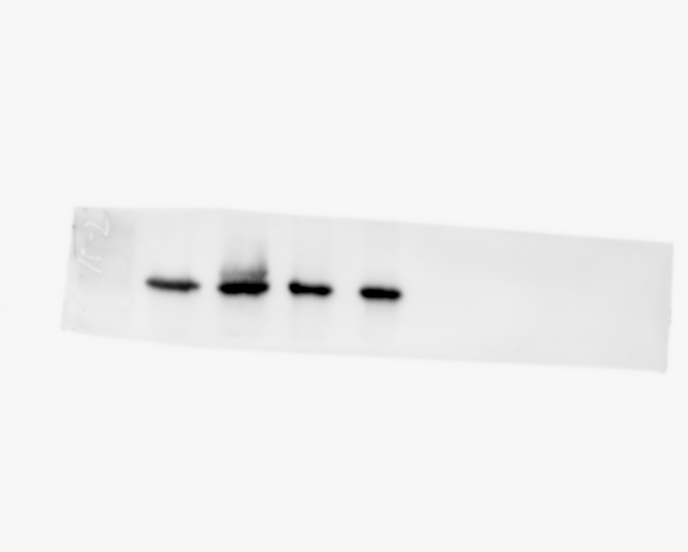

Supplement: Supplementary file 2 [file datasheet2.zip › supplementary material1/figure3D/si-NQO1-cell-1.tif]

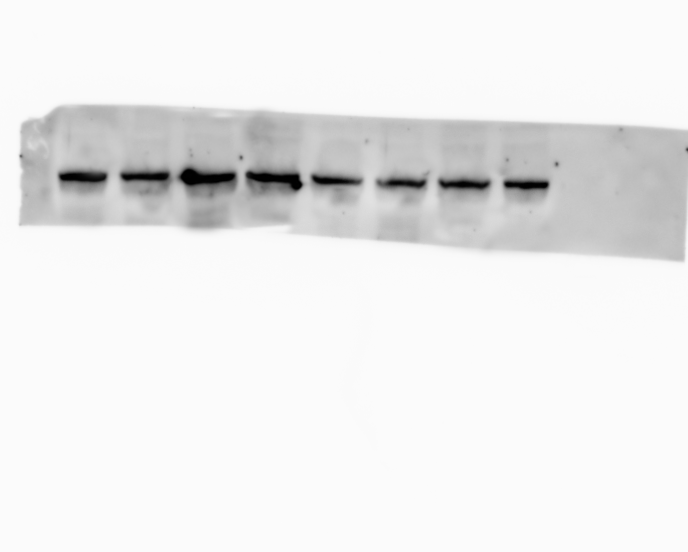

Supplement: Supplementary file 2 [file datasheet2.zip › supplementary material1/figure3D/si-NQO1-cell-2.tif]

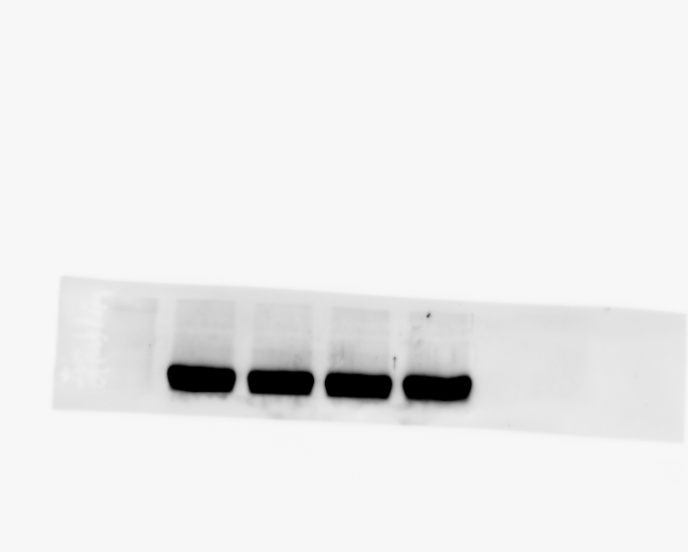

Supplement: Supplementary file 2 [file datasheet2.zip › supplementary material1/figure3D/si-actin-cell-1.tif]

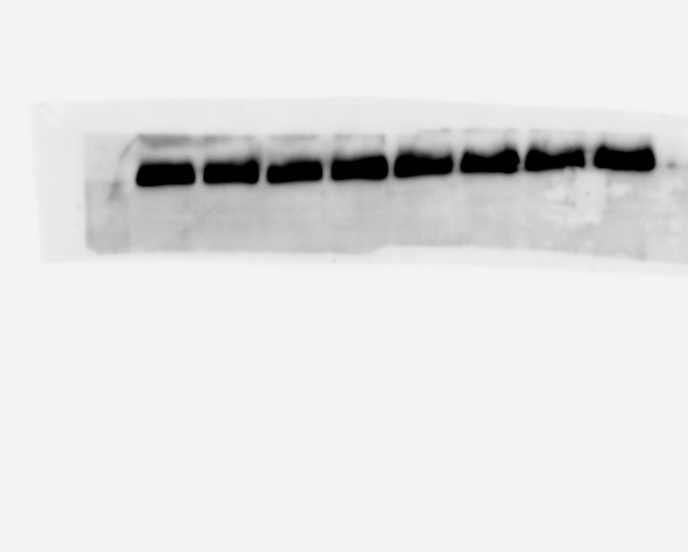

Supplement: Supplementary file 2 [file datasheet2.zip › supplementary material1/figure3D/si-actin-cell-2.tif]
